# Ionically Tunable Gel Electrolytes Based on Gelatin‐Alginate Biopolymers for High‐Performance Supercapacitors

**DOI:** 10.1002/smll.202503937

**Published:** 2025-06-17

**Authors:** Pietro Tordi, Verónica Montes‐García, Adrián Tamayo, Massimo Bonini, Paolo Samorì, Artur Ciesielski

**Affiliations:** ^1^ ISIS UMR7006 Université de Strasbourg, CNRS 8 allée Gaspard Monge Strasbourg F‐67000 France; ^2^ Department of Chemistry “Ugo Schiff” and CSGI University of Florence via della Lastruccia 3 Sesto Fiorentino 50019 Florence Italy

**Keywords:** alginate, crosslinking, energy storage, gelatin, gel electrolytes, organohydrogels, supercapacitors

## Abstract

The development of sustainable, high‐performance gel polymer electrolytes (GPEs) is crucial for next‐generation energy storage; however, existing materials often exhibit limited mechanical stability, suboptimal ionic transport, or environmental drawbacks. Here, for the first time gelatin‐alginate organohydrogels  crosslinked with Cu^2+^ and Mn^2+^ are used as GPEs for supercapacitors, in combination with Li^+^ incorporation to enhance ionic conductivity and transport. Small‐Angle X‐ray Scattering (SAXS) reveals that the choice of the crosslinking cation governs the nanoscale organization of the polymer network—reflected in distinct correlation lengths—which in turn critically influences ionic transport, mechanical stability, and electrochemical performance. Cu^2+^‐crosslinked gels achieve the highest areal capacitance (591.8 mF cm^−2^), energy density (82.2 µWh cm^−2^), and power density (1957.8 µW cm^−2^), whereas Mn^2+^‐crosslinked gels exhibit superior cycling stability (88.3% retention over 5000 cycles). Li^+^ incorporation increases the mechanical flexibility of Mn‐based gels—reducing the compressive modulus by over 60%—enhancing ion mobility and charge storage. Conversely, Cu‐based gels maintain structural integrity while exhibiting improved conductivity. These findings demonstrate how biopolymer‐based GPEs, designed through nanoscale engineering and ion doping, achieve an optimal balance of mechanical robustness and electrochemical performance. By combining scalability and exceptional energy storage capabilities, these materials establish a new paradigm for flexible supercapacitors and sustainable energy technologies.

## Graphical abstract

Gelatin‐alginate organohydrogels crosslinked with Cu^2+^ or Mn^2+^ exhibit distinct nanoscale architectures, as revealed by SAXS, which directly influence ionic transport and electrochemical performance. The interplay between crosslinking and Li⁺ doping modulates network stability and conductivity, providing a rational design strategy for high‐performance gel polymer electrolytes in supercapacitors.

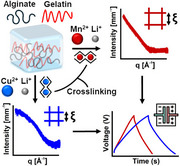

## 1 Introduction

As global energy demand escalates, the development of efficient and sustainable energy storage systems has become increasingly critical.^[^ ^1^, ^2^ ^]^ By closing the gap between batteries and traditional capacitors, supercapacitors have emerged as disruptive technology, offering power densities up to 10 kW/kg and lifespans extending to millions of charge–discharge cycles. Their ability to deliver rapid and intense energy bursts makes them well‐suited for applications in electric vehicles, renewable energy systems, and backup power supplies. Unlike batteries, supercapacitors store energy electrostatically, minimizing degradation and ensuring long‐term stability, making them a compelling alternative in power‐intensive scenarios.^[^ ^3^, ^4^ ^]^

Electrolytes are key components of supercapacitors as they play a pivotal role in determining their energy storage efficiency and device stability.^[^ ^5^ ^]^ Aqueous electrolytes, such as sulfuric acid and potassium hydroxide, display high ionic conductivities and cost‐effectiveness but suffer from corrosion and a restricted voltage window, limiting their energy density. Organic electrolytes, including acetonitrile‐based solutions, allow for higher voltage operation (up to 3 V) yet they pose safety concerns due to flammability and cost. Ionic liquids offer an even wider electrochemical stability range (≈4 V) and excellent thermal stability but their operation is hindered by the material's high cost and viscosity, with the latter determining a power density reduction. Gel polymer electrolytes (GPEs), which combine the advantages of liquid and solid‐state electrolytes, offer enhanced safety and mechanical stability, positioning them as a promising alternative to conventional liquid and solid electrolytes.^[^ ^6^, ^7^ ^]^ Upon immobilizing liquid electrolytes within a polymer matrix, GPEs mitigate issues like electrolyte leakage and ensure mechanical compliance required for modern energy storage technologies.^[^ ^6^, ^7^ ^]^

Among GPEs, biopolymer‐based gel polymer electrolytes (BGPEs) offer a unique synergy of sustainability and electrochemical functionality.^[^ ^8^ ^]^ Conventional hydrogels have been explored as potential BGPEs; however, their susceptibility to water loss under ambient conditions, coupled with suboptimal mechanical properties, call for thoughtful modifications through blending, doping, or network reinforcement.^[^ ^8^ ^]^ Water evaporation not only compromises their mechanical integrity but also impairs ionic transport, limiting their viability for long‐term energy storage applications. Organohydrogels, which combine the hydrophilic nature of hydrogels with enhanced environmental and mechanical stability, effectively overcome these limitations.^[^ ^9^ ^]^ However, their relatively low water content can impede ionic mobility, presenting a fundamental challenge in achieving an optimal balance in electrochemical performance.

Among the employed materials for organohydrogel fabrication, poly(vinyl alcohol) (PVA) is by far the most explored option due to its excellent mechanical properties and stability.^[^ ^10^, ^11^, ^12^ ^]^ Despite its widespread exploitation, PVA's limited biodegradability in natural environments raises concerns regarding its long‐term sustainability.^[^ ^13^, ^14^, ^15^, ^16^, ^17^ ^]^ This underscores the need for alternative materials that offer both performance and environmental benefits. In this context, gelatin and alginate – both derived from renewable resources – have recently emerged as promising candidates. Gelatin, a polypeptide derived from collagen, forms highly elastic and versatile gel networks,^[^ ^18^, ^19^ ^]^ whereas alginate, a polysaccharide extracted from brown algae, is distinguished by its ability to crosslink with divalent cations.^[^ ^20^ ^]^ This crosslinking behavior follows a well‐defined affinity trend^[^ ^20^, ^21^, ^22^, ^23^ ^]^—Pb^2+^ > Cu^2+^ > Cd^2+^ > Ba^2+^ > Sr^2+^ > Ca^2+^ > Co^2+^ ≈ Ni^2+^ ≈ Zn^2+^ > Mn^2+^, enabling precise modulation over gel properties, including mechanical strength and ionic conductivity.^[^ ^20^ ^]^

Building on these principles, in this work we report the first application of a gelatin‐alginate organohydrogel as a gel polymer electrolyte for supercapacitors, offering a fully biopolymeric alternative to conventional PVA‐based systems. What distinguishes this system is not only its green composition, but the ionically tunable network, where Cu^2+^ or Mn^2+^ crosslinking, modulated by Li⁺ incorporation, allows precise control over both mechanical stiffness and ionic transport.

Our approach integrates material sustainability, nanoscale structural tuning, and device‐level optimization within a single platform. This results in exceptional electrochemical performance, with Cu^2^⁺‐crosslinked gels reaching areal capacitance of 591.8 mF/cm^2^, and Mn^2+^‐based gels achieving 88.3% retention over 5,000 cycles.

A direct comparison with state‐of‐the‐art systems demostrates that our materials offer competitive performance in terms of capacitance and energy density. This supports the potential of gelatin–alginate organohydrogels as a promising platform for sustainable, high‐performance gel polymer electrolytes.

## 2 Results and Discussion

**Figure 1:**
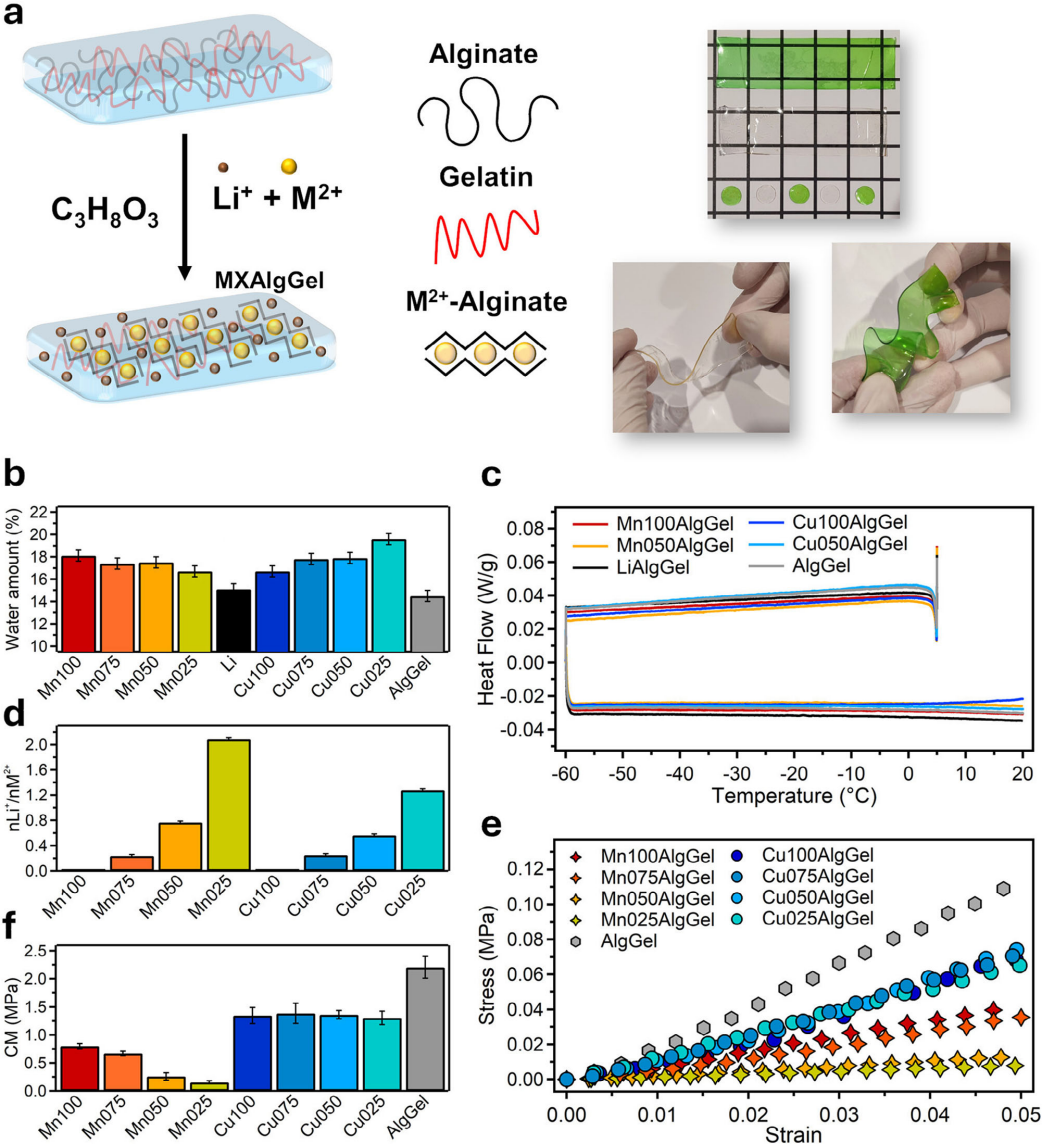
a) Schematic representation of the preparation process for AlgGel‐based organohydrogels and their visual appearance. b) Water content, and c) freezable water evaluation through DSC analysis (Exo up). d) Experimental Li^+^ over M^2+^ molar ratio (nLi^+^/nM^2+^) for the AlgGel‐based organohydrogels. e) Stress‐strain curves, and f) compressive modulus (CM) values of Cu100AlgGel, Cu075AlgGel, Cu050AlgGel, Cu025AlgGel, Mn100AlgGel, Mn075AlgGel, Mn050AlgGel, Mn025AlgGel and AlgGel.

**Figure** **1**a displays the synthetic pathway to form alginate‐gelatin (AlgGel)‐based organohydrogels. The procedure involves the dissolution of gelatin in water at 50 °C, followed by the incorporation of a 4% (w/v) alginate solution under continuous stirring (see experimental section for more details). The resulting mixture is cast into molds, cooled, and subsequently crosslinked in MCl_2_ (M = Mn or Cu) and LiCl solutions. After equilibration in a glycerol‐water solution, the materials are dried at room temperature to yield MXAlgGel samples, where X denotes the molar concentration of MCl_2_, specifically 100 (1.00 M), 075 (0.75 M), 050 (0.50 M), or 025 (0.25 M). The LiCl concentration is adjusted to always maintain a total salt concentration of 1 M across all the formulations, ensuring a systematic modulation of the [Li^+^]/[M^2+^] ratio within the crosslinking solution. This controlled ionic environment enables a precise investigation of the effects of ion composition on the physicochemical and electrochemical properties of the hydrogels (Table S1, Supporting Information). Additionally, LiAlgGel (1.00 M Li) and AlgGel samples serve as reference systems to evaluate the specific influence of metal ions on the organohydrogels. The comparable thickness of all formulations, confirmed in Figure S1 and Table S3 (Supporting Information), ensures a robust framework for performance comparisons.

The incorporation of Mn^2+^ and Cu^2+^ into alginate significantly alters the polymer matrix, restricting chain mobility and inducing water‐insolubility. Qualitatively, Mn^2+^‐crosslinked hydrogels exhibit a softer texture compared to their Cu^2+^‐crosslinked counterparts, consistent with the stronger affinity of Cu^2+^ for alginate.^[^ ^20^ ^]^ On the other hand, MnXAlgGel and LiAlgGel organohydrogels share a similar mechanical consistency. The gels also exhibit distinct optical properties: Mn^2+^‐crosslinked samples appear transparent, whereas Cu^2+^‐crosslinked samples adopt a green hue (Figure 1a; Figure S1d, Supporting Information).

As detailed in Table S3 (Supporting Information), the organohydrogels predominantly consist of glycerol (≈60 wt%), while gelatin and alginate collectively contribute ≈20 wt%. The remaining mass (≈20 wt%) is attributed to water (Figure 1b), as confirmed by Thermogravimetric nalysis (TGA) (Figure S2 and Table S3, Supporting Information), along with homogeneously dispersed ions. The absence of phase separation, combined with glycerol's plasticizing effect, preserves the structural integrity of the gels over several months (Figure S1d–f, Table S2, Supporting Information). Moreover, Differential Scanning Calorimetry (DSC) analysis (Figure 1c) reveals no detectable phase transition during the heating and cooling cycles, indicating that the water within the matrix is predominantly non‐freezable.^[^ ^24^ ^]^ This suggests that the materials retain flexibility and ionic mobility even at subzero temperatures.

The composition of the crosslinking solution, which varies in Li^+^ and Mn^2+^ (or Cu^2+^) molar ratios (Table S1, Supporting Information), dictates the ion incorporation profile of the resulting organohydrogels (Table S4, Supporting Information), As illustrated in Figure 1d, the Li^+^ uptake increases proportionally with its concentration in the crosslinking medium, although its incorporation efficiency differs significantly between Mn^2+^‐ and Cu^2+^‐crosslinked systems. In particular, the experimental molar ratio of Li^+^ to M^2+^ (nLi^+^/nM^2+^) in the hydrogel matrix reaches a maximum of 2.08 for Mn025AlgGel and 1.28 for Cu025AlgGel, underscoring the higher permeability of Mn^2+^‐crosslinked networks to Li^+^ ions. This trend aligns with the structural characteristics observed through SAXS analysis, as discussed later.

Importantly, this controlled variation of ionic composition provides a straightforward and reproducible strategy to modulate the internal structure and functional behavior of the gels. Such systematic tuning of ion uptake within a fully biopolymer‐based system offers a degree of design precision that is still underexplored in the field of sustainable gel polymer electrolytes.

Figure 1e,f displays the compressive behavior of the gels. They reveal that AlgGel, which is characterized by the lowest water content due to the lack of hygroscopic ions, exhibits the highest compressive modulus (CM, ≈2.20 MPa). Among Mn^2+^‐crosslinked gels, Mn100AlgGel attains a CM of ≈0.80 MPa, whereas Mn025AlgGel undergoes a pronounced decline to ≈0.15 MPa, reflecting progressive network disorganization with increasing Li^+^ content. In contrast, Cu^2+^‐crosslinked gels display consistently high CMs (≈1.36 MPa), irrespectively of Li⁺ incorporation. This mechanical robustness is attributed to the strong Cu^2+^‐alginate interactions, which preserve network integrity even in the presence of Li^+^ ions. These findings reveal that, while Li^+^ ions addition destabilizes Mn^2+^‐crosslinked networks, Cu^2+^‐crosslinked systems retain their mechanical strength, making them superior candidates for applications requiring both stability and adaptability.

**Figure 2:**
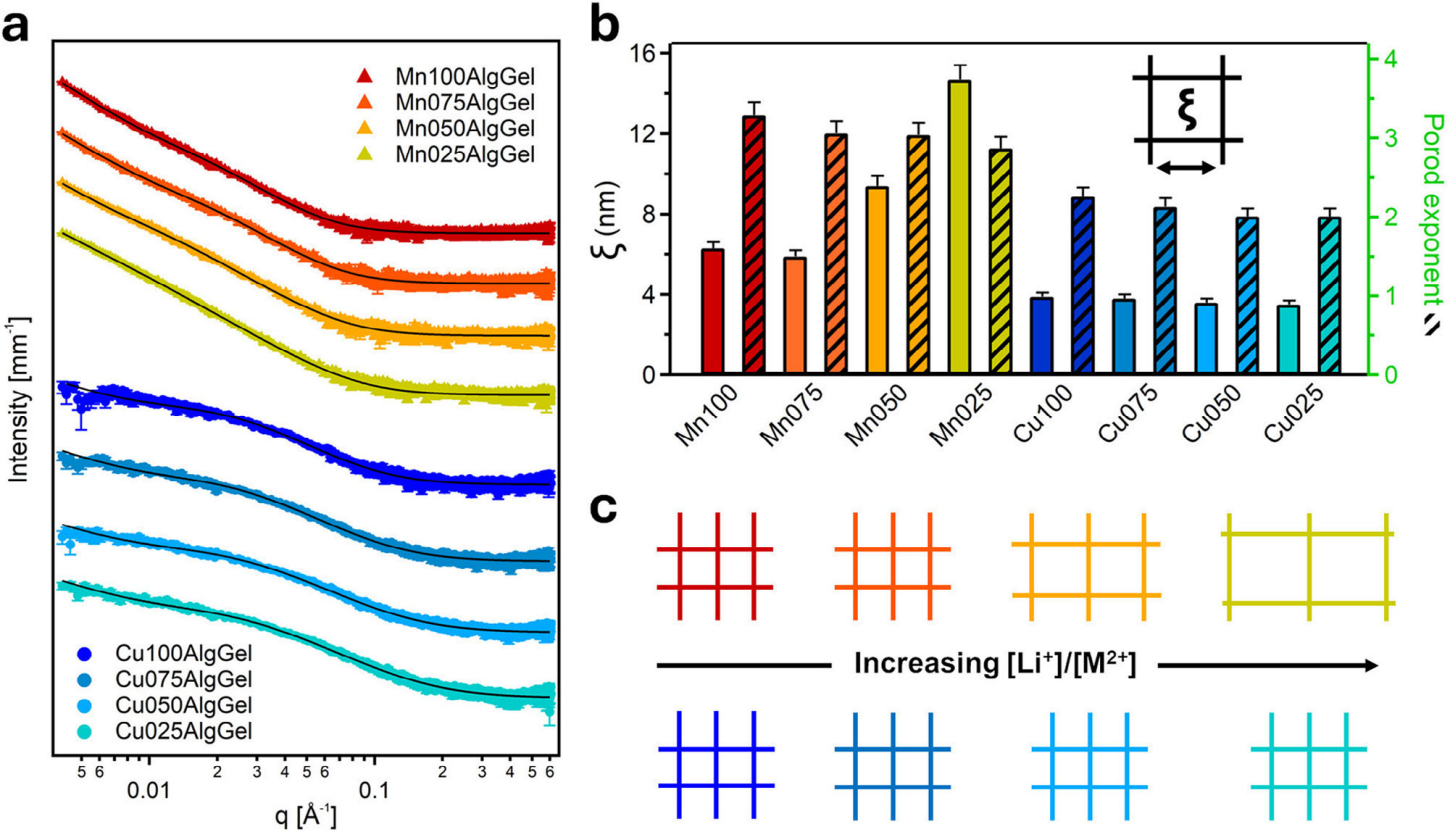
a) SAXS curves (in log scale and offset for clarity) and b) extracted correlation lengths (ξ) and Porod exponents (dashed bars) for MnXAlgGel and CuXAlgGel. c) Schematic representation of the structural differences between Mn^2+^‐ and Cu^2+^‐crosslinked networks as a function of [Li^+^]/[M^2+^] ratio in the crosslinking solution. MnXAlgGels show increasing *ξ* with higher Li^+^ content, indicating a progressively looser network. In contrast, CuXAlgGels maintain a consistently compact network structure.

The structural characteristics of alginate‐gelatin gels crosslinked with Cu^2+^ or Mn^2+^ ions, in combination with Li^+^, offer valuable insights into their potential as GPEs for energy storage applications. SAXS analysis is employed to elucidate how these crosslinking ions govern the nanoscale organization of the gels, thereby establishing a direct correlation between their internal structure, mechanical and functional properties. To quantify the correlation lengths between the polymer chains, SAXS curves (**Figure** **2**a) are fitted using the following equation:^[^ ^25^, ^26^, ^27^, ^28^ ^]^

(1) $$I\left(q\right)=\frac{A}{q^{n}}+\frac{B}{1+{\left(q\xi \right)}^{m}}+bkg$$

in which *A* is the Porod scaling factor, *n* is the Porod exponent, *B* is the Lorentzian scale factor, *m* is the Lorentzian exponent, *ξ* is the correlation length and *bkg* is the background. The results of the fittings are reported in Table S5 (Supporting Information), while correlation lengths and Porod exponents are presented in Figure 2b.

Cu^2+^ and Mn^2+^ ions facilitate alginate crosslinking primarily through interactions with carboxylate groups, though their binding strengths differ significantly. Cu^2+^ establishes stronger and more localized interactions compared to Mn^2+^, resulting in a denser and more rigid hydrogel network.^[^ ^24^, ^29^ ^]^ This difference is apparent in the SAXS analysis, where the correlation lengths (*ξ*) in Cu^2+^‐crosslinked gels are consistently small (3.5–3.9 nm) regardless of the nLi^+^/nCu^2+^ ratio, indicating a structurally resilient network. In contrast, the Mn^2+^‐crosslinked gels exhibit a pronounced increase in *ξ* from 6.3 nm to 14.7 nm when increasing nLi^+^/nMn^2+^ ratios, reflecting the progressive disruption of Mn^2+^ crosslinking by Li^+^ incorporation. This effect can be attributed to competitive ion exchange,^[^ ^20^ ^]^ which leads to a progressively more open network (Figure 2c). In contrast, Cu^2+^‐crosslinked gels maintain their structural integrity, effectively resisting such perturbations.

The Porod exponent (*n*) further provides evidence of these structural differences. In Mn^2+^‐crosslinked gels, *n* decreases from 3.29 to 2.87 as the nLi^+^/nMn^2+^ ratio increases, indicating a transition from a compact, mass‐fractal‐like network to a more open structure.^[^ ^25^, ^26^, ^27^, ^28^ ^]^ In contrast, Cu^2+^‐crosslinked gels exhibit lower and less dissimilar *n* values (2.26 to 2.01), indicative of a surface‐fractal‐like morphology. This subtle yet important distinction emphasizes the rigidity imparted by Cu^2+^ ions, which creates a network resistant to deformation despite appearing less compact at the nanoscale.

This interplay between rigidity and flexibility presents distinct advantages for energy storage applications. The highly crosslinked, structurally robust Cu^2+^‐gel network displays exceptional mechanical stability. Their highly organized nanoscale structure, as observed through SAXS, suggests efficient ion transport pathways, making them strong candidates for applications requiring robust and stable electrolyte frameworks, such as solid‐state batteries. Conversely, Mn^2+^‐crosslinked gels feature looser and more flexible networks with the increase of Li^+^ ions concentration, providing enhanced ionic mobility due to their porous architecture. While this increased mobility, later confirmed by electrochemical characterization, may offer advantages in facilitating ion movement, the weaker crosslinking interactions could compromise long‐term mechanical resilience. By modulating Cu^2+^, Mn^2+^, and Li^+^ ratios, these gels can be engineered to achieve an optimal balance between mechanical robustness and ionic conductivity. Notably, this dual‐ion crosslinking approach – combined with SAXS as a structural diagnostic – offers a rare opportunity to deliberately tune nanoscale gel architecture in fully biopolymer‐based systems. This approach enables precise control over internal organization, directly linking nanoscale structure to electrochemical performance, a crucial but often overlooked factor in the design of next‐generation sustainable gel polymer electrolytes.

This versatility underscores their potential as next‐generation GPEs, with SAXS providing a critical foundation for understanding and optimizing their performance.

To evaluate the thermal stability of AlgGel‐based gel electrolytes for energy storage applications, TGA was conducted. Figure S2 and Table S3 (Supporting Information) reveal that all samples undergo thermal degradation onset at approximately 190 °C, except for those with high Cu^2+^ content (Cu075–Cu100), which degrade at slightly lower temperatures (≈180 °C) due to the catalytic effect of Cu^2+^ on decomposition.^[^ ^20^, ^30^ ^]^ These results confirm that, despite their biopolymeric nature, gelatin‐alginate gels maintain the thermal tolerance required for practical energy storage applications, where organohydrogel electrolytes are generally expected to operate at temperatures up to approximately 80 °C.^[^ ^8^, ^31^, ^32^ ^]^

Building on their distinct physicochemical properties, we assess the electrochemical performance of AlgGel‐based organohydrogels as gel electrolytes in symmetric supercapacitors, establishing correlations with their nanoscale structures as unveiled by SAXS. To this end, coin‐cells were assembled using activated carbon as both the cathode and anode materials, while the different organohydrogel formulations served as electrolytes (see experimental section for more details). Their electrochemical properties were systematically analyzed to determine their viability for energy storage applications.

**Figure 3:**
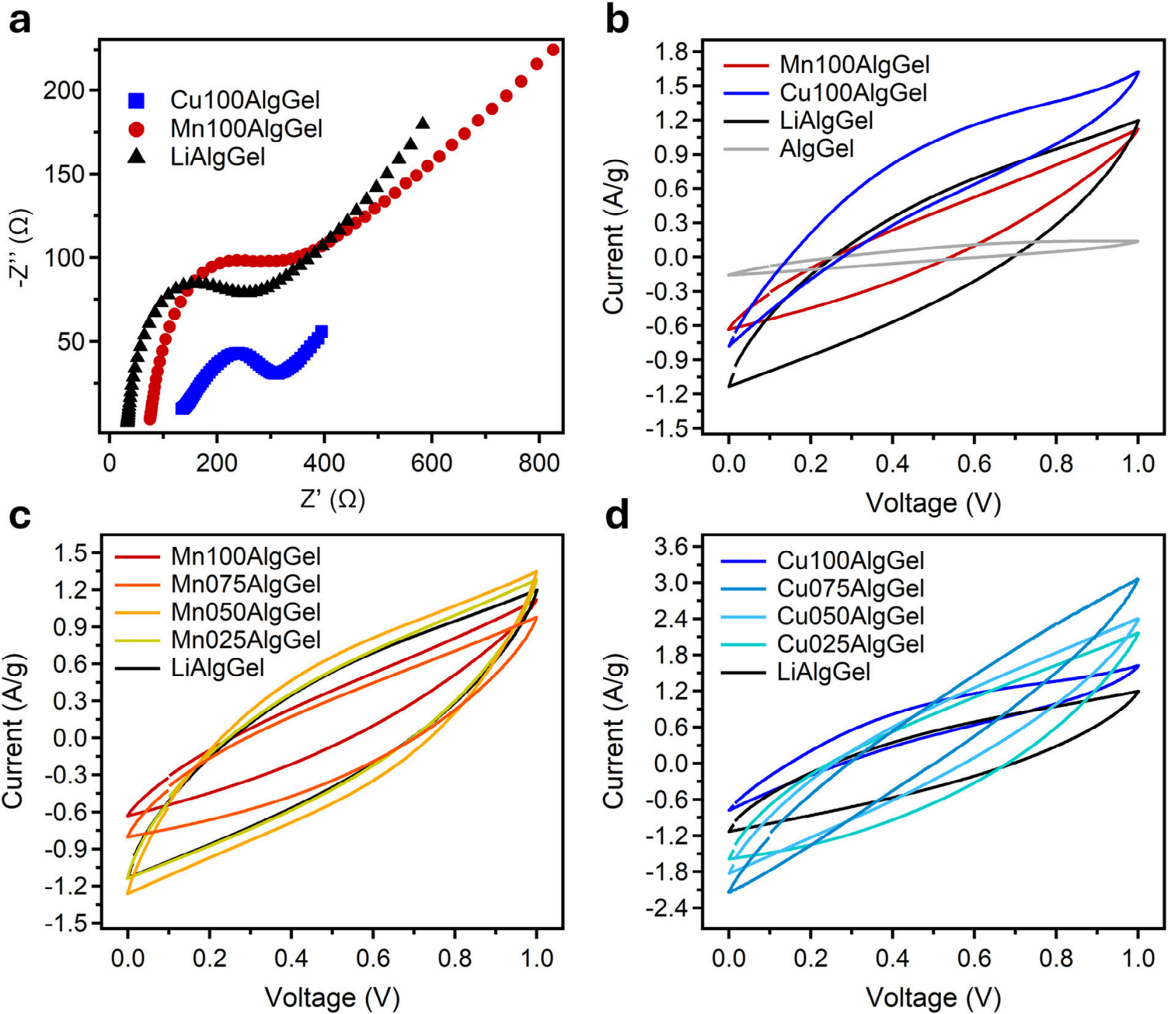
a) Nyquist plot showing the impedance behavior for Cu100AlgGel, Mn100AlgGel, and LiAlgGel in supercapacitors. A comparison with AlgGel is provided in Figure S3a (Supporting Information). b) CV curves of gels with single cationic species at 10 mV s^−1^ in supercapacitors. CV curves at 10 mV s^−1^ of c) MnXAlgGels and d) CuXAlgGels in supercapacitors. The CV curves for other scan rates are reported in Figures S4–S6 (Supporting Information).

Electrochemical impedance spectroscopy (EIS) was conducted to analyze the ionic transport characteristics (**Figures** **3**a and S3, Supporting Information). A key parameter extracted from EIS is the bulk resistance (R_b_), which accounts for intrinsic electrolyte resistance, including ion migration within the gel matrix. In Nyquist plots, R_b_ is identified as the high‐frequency intercept on the real axis (Z'), reflecting the ease with which ions move within the electrolyte. For gel electrolytes, low bulk resistance is crucial, as it indicates efficient ionic conductivity and minimal energy loss during charge transport. It is important to note that the gels are electronically insulating; therefore, the conductivity values extracted from EIS (see Table S6, Supporting Information) refer exclusively to ionic transport. Elevated R_b_ may indicate hindered ion mobility due to factors such as poor ion dissociation, increased viscosity, or excessive crosslinking density within the gel network. Another critical parameter, the charge transfer resistance (R_ct_), quantifies the efficiency of ion exchange at the electrode‐electrolyte interface, where lower R_ct_ values indicate faster electrochemical kinetics, enabling rapid charge/discharge cycles, which is essential for high‐power applications.

Among gels crosslinked exclusively with single divalent metal ions, Cu100AlgGel exhibits the highest R_b_ (119.0 Ω, Table S6, Supporting Information), consistent with its tightly packed and localized Cu^2+^‐alginate network as revealed by SAXS (ξ = 3.9 nm). In contrast, Mn100AlgGel shows a lower R_b_ (73.3 Ω) due to its looser and more open structure (ξ = 6.3 nm), which facilitates faster ion diffusion through the bulk. However, the charge transfer resistance (R_ct_) displays the opposite trend: Cu100AlgGel has a lower R_ct_ (239.9 Ω) compared to Mn100AlgGel (296.4 Ω). This suggests that the stronger coordination of Cu^2+^ with the alginate matrix forms a more cohesive and stable electrode–electrolyte interface, promoting efficient ion–electron exchange. Conversely, the weaker and more dynamic Mn^2+^–polymer interactions, while favorable for bulk transport, may lead to a less well‐defined interfacial structure and hinder charge transfer. These results demonstrate that R_b_ and R_ct_ capture different aspects of the electrochemical behavior – bulk conductivity and interfacial kinetics, respectively – and must be interpreted independently when evaluating gel electrolyte performance. LiAlgGel, with its highly disordered and porous matrix, exhibits the lowest R_b_ (32.7 Ω), indicating superior bulk ion mobility. Notably, as shown in Figure S3b, c (Supporting Information), even small additions of Li^+^ in Mn^2+^‐ and Cu^2+^‐crosslinked gels (Mn075AlgGel and Cu075AlgGel) significantly reduce R_b_, making their impedance profiles increasingly similar to that of LiAlgGel.

Cyclic voltammetry (CV) measurements (Figure 3b) provide further insights into the electrochemical performance of the gel electrolytes. The area enclosed by the CV loop is directly proportional to the material's capacitance and overall electrochemical performance, with larger areas indicating greater charge storage capacity and improved ionic mobility. Among the samples, LiAlgGel displays the largest CV loop, demonstrating its high capacitance, which arises from its open structure and excellent ionic transport properties. In contrast, Cu100AlgGel and Mn100AlgGel display smaller loop areas due to the strong M^2+^‐polymer interactions that reinforce the polymer matrix. While these interactions enhance structural stability, they also restrict ionic mobility, leading to lower capacitances. As expected, AlgGel, lacking crosslinked metal ions, exhibits negligible electrochemical activity.

Figure 3c,d further illustrate the impact of Li^+^ incorporation on electrochemical performance. Increasing the nLi^+^/nCu^2+^ ratio progressively expands the CV loop area, as Li^+^ ions disrupt the Mn^2+^‐dominated network, enhancing ionic mobility and increasing capacitance, while simultaneously reducing structural rigidity. In the Cu^2+^‐crosslinked gels, higher nLi^+^/nCu^2+^ ratio yields a synergistic effect, where Li^+^ ions improve ion transport while Cu^2+^ ions preserve structural integrity. This combination results in progressively larger CV loops, with Cu050AlgGel surpassing even LiAlgGel, demonstrating its high potential for energy storage applications.

**Figure 4:**
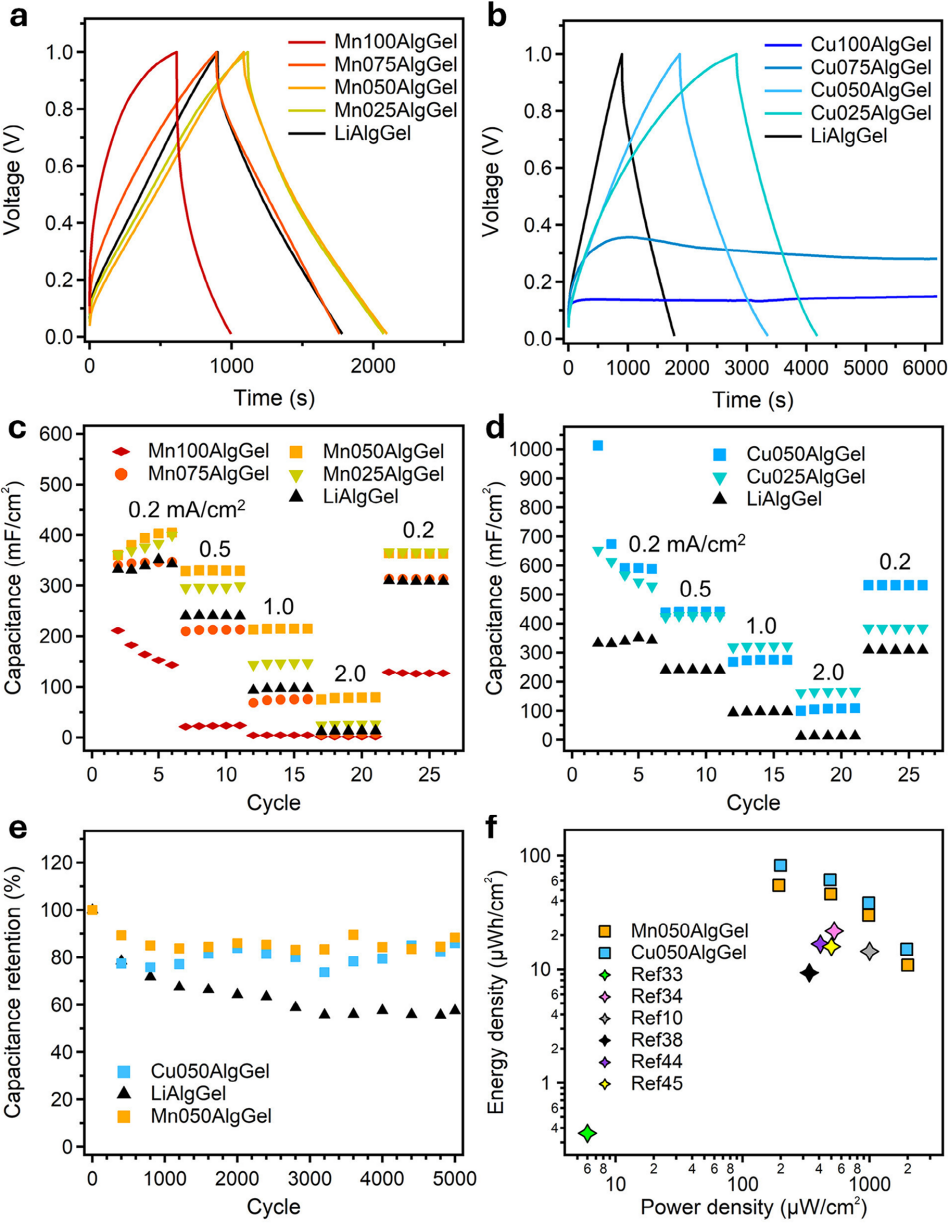
GCD curves of a) MnXAlgGels and b) CuXAlgGels along with LiAlgGel. (current density = 0.2 mA/cm^2^, other current densities can be seen in Figures S7,S8, Supporting Information). Figure S9 (Supporting Information) compares the GCD curves of AlgGel, Mn100AlgGel, Cu100AlgGel and LiAlgGel. In panel c) and d) the areal capacitances of MnXAlgGels and CuXAlgGels (respectively) along with LiAlgGel, at different current densities (*i.e*., 0.2 mA cm^−2^, 0.5 mA cm^−2^, 1.0 mA cm^−2^, 2.0 mA cm^−2^). Both Mn‐ and Cu‐based systems exhibit excellent rate capability, as the capacitance measured at 0.2 mA cm^−^^2^ is fully recovered and remains stable after cycling at a high current density of 2.0 mA cm^−^^2^, indicating robust structural and electrochemical reversibility. e) Capacitance retention over cycling of Mn050AlgGel, Cu050AlgGel and LiAlgGel over 5000 charge/discharge cycles. f) Ragone plot comparing the energy and power densities of Mn050AlgGel and Cu050AlgGel with state‐of‐the‐art references from Table S9 (Supporting Information).

The galvanostatic charge‐discharge (GCD) profiles (**Figure** **4**a,b) provide further insights into the electrochemical performance of Mn^2+^‐ and Cu^2+^‐crosslinked organohydrogel electrolytes, revealing their charge storage capabilities and stability. The shape and duration of the charge‐discharge profiles reveal key differences in ionic transport and capacitance retention across the different formulations. The GCD curves of MnXAlgGels show a progressive improvement in charge storage when increasing the nLi^+^/nMn^2+^ ratio. Mn050AlgGel exhibits the longest discharge time, confirming its optimal balance between structural stability and enhanced ionic mobility. This behavior aligns with the CV results, where Mn050AlgGel demonstrated a larger electrochemical response. In contrast, Mn100AlgGel shows a significantly shorter discharge time, indicating higher internal resistance, and limited ionic transport due to its more rigid Mn^2+^‐dominated network, in agreement with EIS and SAXS characterization, respectively. The gradual transition from Mn100AlgGel to Mn025AlgGel reveals how Li^+^ incorporation progressively disrupts the Mn^2+^ network, improving charge storage efficiency in full agreement with the previous characterizations.

The GCD curves for CuXAlgGels display notable differences in performance, reflecting the impact of Cu^2+^ crosslinking on ion mobility and capacitance. Cu050AlgGel outperforms the other Cu‐based gels, exhibiting the longest discharge time, consistent with its superior capacitance observed in CV measurements (see Figure 3d). The synergy between Cu^2+^ and Li^+^ optimizes both structural integrity and ionic transport, making Cu050AlgGel a promising candidate for energy storage applications. However, Cu100AlgGel and Cu075AlgGel struggle to reach 1 V, which suggests high internal resistance due to excessively strong Cu^2+^‐polymer interactions restricting ion mobility. This observation is further supported by the EIS data, where Cu100AlgGel exhibits the highest R_b_. LiAlgGel, despite its high ionic mobility, shows a shorter discharge duration compared to Mn050AlgGel and Cu050AlgGel, reinforcing the idea that, while Li^+^ enhances ion transport, the absence of divalent metal crosslinking reduces structural stability and long‐term performance.

Figure 4c,d and Table S7 (Supporting Information) display the areal capacitance of AlgGel‐based organohydrogels, highlighting the impact of crosslinking composition on ionic transport and charge storage. In Mn^2+^‐crosslinked gels (Figure 4c), capacitance improves with increasing the nLi^+^/nMn^2+^ ratio, peaking at Mn050AlgGel (394.2 mF/cm^2^ at 0.2 mA cm^−2^). This balance optimizes ionic mobility while maintaining structural integrity. In Cu^2+^‐crosslinked gels (Figure 4d), Cu050AlgGel achieves the highest capacitance (591.8 mF cm^−2^ at 0.2 mA cm^−2^), surpassing both Mn‐based gels and LiAlgGel. This outstanding performance arises from the synergistic effect of Cu^2+^ maintaining network stability while Li^+^ enhances ion transport. SAXS and mechanical data confirm that the compact and stable Cu^2+^ network supports efficient ionic transport even with increasing Li^+^ content.

The capacitance retention of Mn050AlgGel, Cu050AlgGel, and LiAlgGel over 5000 charge‐discharge cycles (Figure 4e) provides further insights into their long‐term electrochemical performance. Among the samples, Mn050AlgGel exhibits the highest capacitance retention (Figure 4e), maintaining 88.3% of its initial value (from 404.68 to 357.27 mF cm^−2^) over 5000 charge/discharge cycles, highlighting its robust structural integrity and sustained ionic mobility over extended cycling. Cu050AlgGel follows closely, with 85.9% capacitance retention (from 588.6 to 505.56 mF cm^−2^), reinforcing the mechanical resilience of Cu^2+^ crosslinking in preventing degradation while maintaining efficient charge storage. In contrast, LiAlgGel exhibits the lowest capacitance retention (≈57.6%, from 342.76 to 197.38 mF cm^−2^), which we attribute to minor interfacial rearrangements during extended cycling. This behavior is expected in non‐crosslinked systems and does not reflect any change in composition or chemical stability, as confirmed by XPS analysis.

The ionic composition of Mn050AlgGel and Cu050AlgGel remains stable after prolonged cycling, as confirmed by XPS survey spectra (Figures S10a,S11a, Supporting Information) and the corresponding elemental analysis (Table S8, Supporting Information), which reveal no significant changes in atomic ratios. High‐resolution Mn 2p and Cu 2p spectra (Figures S10c,d and S11c,d, Supporting Information) confirm the retention of the Cu^2+^ oxidation state and indicate the presence of partially oxidized Mn^3+^, likely arising from surface oxidation of Mn^2+^ during sample handling or XPS measurement. Notably, these oxidation features remain unchanged after 5000 charge/discharge cycles, demonstrating redox stability. The gels also retain their macroscopic appearance (Figure S10b,S11b, Supporting Information), indicating structural integrity. These results demonstrate that the excellent electrochemical performance is primarily governed by the intrinsic chemical and physical stability of the ionically crosslinked biopolymer network.

Table S9 (Supporting Information) and Figure 4f demonstrate that Cu050AlgGel and Mn050AlgGel outperform most state‐of‐the‐art organohydrogel electrolytes in capacitance, energy density, and power density, establishing them as highly promising materials for sustainable energy storage. Cu050AlgGel achieves the highest capacitance (591.8 mF cm^−2^, 59.2 F g^−1^) and energy density (82.2 µWh cm^−2^, 8.2 Wh kg^−1^), surpassing conventional hydrogel‐based electrolytes, while Mn050AlgGel follows closely (capacitance: 394.2 mF cm^−2^, 39.4 F g^−1^; energy density: 54.8 µWh cm^−2^, 5.5 Wh kg^−1^). Their exceptional power densities (1957.8 µW cm^−2^, 196.5 W kg^−1^ for Cu050AlgGel and 1994.9µW cm^−2^, 192.7 W kg^−1^ for Mn050AlgGel) highlight their potential for high‐power applications requiring rapid charge/discharge cycles. Additionally, Mn050AlgGel exhibits the best long‐term stability (88.3% retention over 5000 cycles), while Cu050AlgGel retains 85.9%, reinforcing their durability. These results reflect the effectiveness of our ionically tunable design strategy, where compositional control and nanoscale structural optimization translate directly into device‐level performance. Compared to existing materials, the gelatin‐alginate gels developed in this work present an optimal balance between ionic conductivity, mechanical stability, and electrochemical performance, positioning them as strong candidates for next‐generation, sustainable energy storage solutions.^[^ ^10^, ^11^, ^12^, ^33^, ^34^, ^35^, ^36^, ^37^, ^38^, ^39^, ^40^, ^41^, ^42^, ^43^, ^44^, ^45^ ^]^

## 3 Conclusion

This study demonstrates the successful development of gelatin‐alginate organohydrogels as gel polymer electrolytes for supercapacitors, leveraging divalent metal crosslinking and Li^+^ doping to modulate their mechanical and electrochemical properties. Small‐angle X‐ray scattering provided fundamental insights into how nanoscale structural organization governs ionic transport and mechanical stability, shedding light on the electrochemical behavior of these materials.

The incorporation of Li^+^ significantly influences both mechanical properties and ionic transport in Mn^2+^‐ and Cu^2+^‐crosslinked gels. In Mn^2+^‐crosslinked gels, Li^+^ disrupts the rigid network, reducing the compressive modulus (Mn050AlgGel: ≈0.26 MPa) while enhancing ion mobility, leading to improved charge storage (394.2 mF/cm^2^), an energy density of 54.8 µWh cm^−2^, a power density of 1994.9 µW cm^−2^, and excellent capacitance retention of 88.3% over 5000 cycles. In contrast, Cu^2+^‐crosslinked gels maintain structural integrity (≈1.36 MPa) while benefiting from Li⁺‐induced conductivity improvements, resulting in the highest capacitance (591.8 mF cm^−2^), along with an energy density of 82.2 µWh cm^−2^, a power density of 1957.8 µW cm^−2^, and capacitance retention of 85.9% over 5000 cycles. While Mn‐based gels become more flexible with Li^+^, Cu‐based gels balance mechanical stability and ionic transport, making Li^+^ a crucial factor in optimizing gel polymer electrolytes for high‐performance and durable supercapacitors.

These findings establish gelatin‐alginate gels as promising sustainable candidates for energy storage applications, particularly in flexible electronics, wearable devices, and renewable energy systems. The scalability and environmental sustainability of gelatin and alginate position them as viable alternatives to conventional synthetic electrolytes, supporting industrial adoption. More broadly, this work introduces a rational design strategy for biopolymer‐based electrolytes, where multivalent ionic crosslinking and nanoscale structural tuning are used to control functional performance. This platform may be extended to other soft electrochemical systems, bridging the gap between sustainable materials and application‐specific engineering.

## 4 Experimental Section

### Materials

Sodium alginate (alginic acid sodium salt) was obtained from Sigma‐Aldrich (Product code: 180947, Mannuronate/Guluronate ratio = 1.50 ± 0.04, determined via FT‐IR spectroscopy;^[^ ^46^ ^]^ molecular weight ≃132 kDa, determined via viscosimetric analysis).^[^ ^46^ ^]^ Gelatin derived from porcine skin (Product code: G2625, type A, gel strength ≈175 g Bloom) and glycerol (BioXtra, ≥99%) were also sourced from Sigma‐Aldrich. Manganese chloride tetrahydrate (MnCl_2_·4H_2_O, ACS reagent ≥ 99.8%), lithium chloride (LiCl, ACS reagent ≥ 99.0%), and copper chloride dihydrate (CuCl_2_·2H₂O, ACS reagent ≥ 99.0%) were purchased from Sigma‐Aldrich. Polyvinylidene fluoride (PVDF) binder, and N‐Methyl‐2‐pyrrolidone (NMP) were purchased in Sigma Aldrich, high‐surface active carbon (AC) for super‐capacitor electrode and Super P Conductive Carbon Black were purchased in MTI corporation. Deionized water was used for all solution preparations. All reagents were used without further purification.

### Gel Polymer Electrolytes Preparation

To prepare the gel polymer electrolytes, 1.36 g of gelatin was dissolved in 11.3 mL of water under gentle stirring at 50 °C for 10 minutes. A 4% (w/v) alginate solution (3.80 g) was then added, and the mixture was stirred at 50 °C for an additional 10 minutes.

Next, 6 mL of the solution was poured into polystyrene molds (2 × 6 cm) and allowed to cool at room temperature for 10 minutes. The molds were subsequently refrigerated at 4 °C for 10 minutes and immersed in 50 mL of an aqueous solution containing MCl_2_ (M = Mn or Cu) and LiCl for 1 hour. The specific concentrations of MCl_2_ and LiCl used in the crosslinking solutions were detailed in Table S1 (Supporting Information).

The molds were then transferred to a glycerol:water solution (1:4 by volume) containing the same salt concentrations as detailed in Table S1 (Supporting Information) and incubated for 1.5 hours. To remove excess salts, the molds were immersed in a fresh glycerol:water solution (1:4 by volume) for an additional 1.5 hours. The samples, dried overnight under a fume hood and equilibrated at 40% relative humidity (RH), were named MXAlgGel, where M refers to the metal ion (Mn, or Cu) and X refers to their molar concentration 100 (1.00 M), 075 (0.75 M), 050 (0.50 M), or 025 (0.25 M). Note that the LiCl concentration was adjusted accordingly to always maintain a total salt concentration of 1 M (see Table S1, Supporting Information). LiAlgGel was also studied to understand the effect of the metal ion in the physicochemical properties and electrochemical performance of the resulting organohydrogels. AlgGel was used as a blank for comparative purposes. All samples were stored in hermetically sealed Petri dishes under dark conditions until their use. The results labeled as 6‐month‐old samples were obtained from samples stored according to this protocol. Before characterization, samples were cut into disk shapes or stripes (1 cm diameter and width, respectively).

### Coin‐Cell Assembly

Working electrodes were prepared by mixing AC (80 wt%, 16 mg), Super P (10 wt%, 2 mg), and PVDF (10 wt%, 2 mg) in an agate mortar with several drops of NMP to form a homogeneous paste. The paste was coated onto carbon paper electrodes (0.8 cm diameter) and dried overnight under vacuum at 80 °C. The mass loading of active carbon on each electrode was ≈2 mg·cm^2^. Two electrodes were assembled in CR2032 stainless steel coin cells with the AlgGel‐based organohydrogels (1.0 cm diameter) serving as the electrolyte.

### Characterization Techniques

*Small Angle X‐ray Scattering (SAXS)*: Small angle scattering measurements were performed using a Xeuss 3HR system with a scatterless collimation camera and a hybrid detector (Eiger 1M, Dectris). Cu Kα radiation (λ = 1.542 Å) was produced by an ultra‐brilliant point microfocus X‐ray source (GENIX‐Fox 3D, Xenocs, Grenoble) operating at 30 W. Measurements were taken at two sample‐to‐detector distances (375 mm and 1750 mm) to cover a q‐range of 0.004 Å⁻¹ to 0.6 Å⁻¹. Data analysis was conducted using SasView software. (http://www.sasview.org/).

*Inductively Coupled Plasma‐Atomic Emission Spectrometry (ICP‐AES)*: The Li^+^, Na^+^, Mn^2+^, and Cu^2+^ content in the AlgGel‐based samples was quantified using an Agilent 720‐ES ICP‐AES instrument equipped with a pneumatic nebulizer. Approximately 10 mg of each sample was dissolved in 2 mL of 0.05 M Na_2_EDTA and 10% (w/v) NaOH solution at 80 °C for 1 hour with magnetic stirring. After dilution, measurements were performed in triplicate using calibration lines from certified standards.

*Thermogravimetric Analysis (TGA)*: A Discovery SDT650 (TA Instruments) was used. Samples were analyzed under air flow (100 mL·min^−1^) with a heating rate of 10 °C·min^−1^ from room temperature to 950 °C and using aluminum pans. For the water content determination, a heating ramp from RT to 120 °C (rate of 10 °C·min^−1^), followed by a 10 minutes isotherm. Measurements were performed under N_2_ flux (100 mL·min^−1^), using aluminum pans. Data were analyzed with Trios v5.1.1.46572 software (TA Instruments). Samples were all equilibrated at RH = 40% prior the measurement.

*Differential Scanning Calorimetry (DSC)*: DSC measurements were performed using a Discovery DSC 2500 instrument. Samples equilibrated at 40% RH were hermetically sealed within Tzero Aluminum Hermetic pans (TA Instruments) and then investigated by cooling from 5 °C to −60 °C, then heated from −60 °C to 25 °C, in both cases using a heating rate of 1 °C min^−1^ under nitrogen (N_2_) flow. Data were processed with Trios v5.1.1.46572 software (TA Instruments).

*Mechanical Properties*: Compressive properties AlgGel‐based electrolyte disks (1 cm of diameter) were measured using a Discovery DHR‐3 rheometer, using a head plate with 2 cm of diameter. The compression speed was 2 µm s^−1^. The compressive modulus (CM) of the samples was determined from the slope of the linear fit to the stress – strain curves shown in Figure 1e, within the 0.00 – 0.05 strain range. Traction tests on Mn050AlgGel and Cu050AlgGel samples, before and after storage, were performed using a Mark‐10 M7‐025E digital force gauge mounted on a Mark‐10 ESM‐303E motorized test stand. Samples were cut into stripes (1 cm width), clamped at the edges and stretched at a constant rate of 30 mm/min. The Young's modulus (YM) was determined by linear fitting of the stress vs. strain curves shown in Figure S1f (Supporting Information), within the 0.0 – 0.2 strain range.

*Electrochemical Characterization*: The electrochemical performance of the AlgGel‐based organohydrogels was studied using cyclic voltammetry (CV), and electrochemical impedance spectroscopy (EIS) on Autolab PGSTAT128N Potentiostat / Galvanostat instruments with a Metrohm Autolab DuoCoin Cell Holder (Metrohm AG) at room temperature. CV was performed at scan rates of 2–10 mV s^−1^ in the voltage range between 0 and 1.8 V or 0 and 1.0 V. EIS measurement was recorded with a frequency range of 0.01 Hz to 1 MHz, with data fitted to an equivalent circuit model (Figure S12, Supporting Information) to extract key electrochemical parameters. The galvanostatic charge‐discharge (GCD) tests were carried out on Neware Battery Tester (BTS‐4008T‐5V/10mA, Neware Technology Company, Guangdong, China). GCD curves were tested at current densities ranging from 0.2 to 2 mA cm^−2^.

‐ Calculation of ionic conductivity (σ)

(2) $$\sigma =\frac{l}{R_{b}\cdot A}$$

Being l the gel thickness (Table S3, Supporting Information), A the gel area (0.50 cm^2^) and R_b_ the bulk resistance

*X‐ray Photoelectron Spectroscopy*: X‐ray Photoelectron Spectroscopy (XPS) (Thermo Scientific K‐Alpha X‐ray photoelectron spectrometer) equipped with an aluminum X‐ray source (energy 1.4866 keV) at a vacuum level of 10^−8^–10^−9^ mbar in the main chamber. The spot size of the X‐ray beam was fixed at 400 µm.

## Conflict of Interest

The authors declare no conflict of interest.

## Author Contributions

P.T., V.M.G., M.B., P.S., and A.C. conceived the experiments and designed the study. P.T. performed the physicochemical characterization. P.T. and V.M.G. performed the electrochemical characterization. A.T. measured the electrical conductivity of the gels. P.T., V.M.G., A.T., M.B., P.S., and A.C. discussed the results and contributed to the interpretation of data. P.T., V.M.G., M.B., P.S., and A.C. cowrote the paper with input from all coauthors.

## Supporting information



Supporting Information

## Data Availability

The data that support the findings of this study are available from the corresponding author upon reasonable request.
