# Supplementary material for: Ionically Tunable Gel Electrolytes Based on Gelatin‐Alginate Biopolymers for High‐Performance Supercapacitors
Source: Small. 2025 Jun 17;21(33):2503937. doi: 10.1002/smll.202503937 (PMC12372438; doi:10.1002/smll.202503937)
Supplement: Supplementary file 1 — Supporting Information [file SMLL-21-2503937-s001.docx]

Supporting Information

**Ionically Tunable Gel Electrolytes Based on Gelatin-Alginate Biopolymers for High-Performance Supercapacitors**

*Pietro Tordi, Verónica Montes-García, Adrián Tamayo, Massimo Bonini*, Paolo Samorì*, Artur Ciesielski**

P. Tordi, Dr. V. Montes-García, Dr. A. Tamayo, Dr. A. Ciesielski, Prof. Dr. P. Samorì

University of Strasbourg, CNRS, ISIS UMR 7006, 8 allée Gaspard Monge, F-67000 Strasbourg, France

Email: ciesielski@unistra.fr, samori@unistra.fr

P. Tordi, Prof. Dr. M. Bonini

Department of Chemistry “Ugo Schiff” and CSGI, University of Florence, via della Lastruccia 3, Sesto Fiorentino, Florence, 50019 Italy

Email: massimo.bonini@unifi.it

**Table of Contents**

**Table S1.** Preparation conditions of MXAlgGel polymer electrolytes

**Figure S1.** Optical microscopy images for thickness evaluation, visible light absorption and mechanical stability.

**Table S2.** Traction test results for Mn050AlgGel and Cu050AlgGel, before and after prolonged storage.

**Table S3.** General properties of the MAlgGel electrolytes

**Figure S2.** TGA thermograms for MAlgGel water content and degradative profile evaluation

**Table S4.** Cations’ content of the MXAlgGel samples determined through ICP-AES.

**Table S5.** SAXS analysis fitting parameters

**Figure S3.** Impedance spectra of Cu100AlgGel, Mn100AlgGel, LiAlgGel and AlgGel, Cu075AlgGel, Mn075AlgGel.

**Table S6.** Bulk resistance (R_b_) and charge transfer resistance (R_ct_) values for Mn100AlgGel, Mn075AlgGel, LiAlgGel, Cu100AlgGel, Cu075AlgGel and AlgGel.

**Figure S4.** Cyclic voltammetry curves of AlgGel, Mn100AlgGel, Cu100AlgGel and LiAlgGel at different scan rates

**Figure S5.** Cyclic voltammetry curves of Mn100AlgGel, Mn075AlgGel, Mn050AlgGel and Mn025AlgGel at different scan rates

**Figure S6.** Cyclic voltammetry curves of Cu100AlgGel, Cu075AlgGel, Cu050AlgGel and Cu025AlgGel at different scan rates

**Figure S7.** GCD curves of Mn100AlgGel, Mn075AlgGel, Mn050AlgGel and Mn025AlgGel at different current densities

**Figure S8.** GCD curves of Cu100AlgGel, Cu075AlgGel, Cu050AlgGel and Cu025AlgGel at different current densities

**Figure S9.** GCD curves of AlgGel, Mn100AlgGel, Cu100AlgGel and LiAlgGel at different current densities

**Table S7.** Areal and specific capacitance of Mn050AlgGel, Cu050AlgGel and LiAlgGel at different current desities

**Figure S10.** Mn050AlgGel XPS and morphological characterization before and after 5000 charge-discharge cycles.

**Figure S11.** Cu050AlgGel XPS and morphological characterization before and after 5000 charge-discharge cycles.

**Table S8.** Elemental composition of Mn050AlgGel and Cu050AlgGel before and after 5000 charge–discharge cycles.

**Figure S12.** The equivalent electric circuit models used for fitting the Nyquist plots.

**Table S9.** Summary of the state-of-the-art capacitance performance of organohydrogel electrolytes for energy storage applications

**Figure S13.** Thermogravimetric analysis (TGA) of Mn050AlgGel and Cu050AlgGel samples after 5000 charge/discharge cycles.

**Table S10.** Water content of Mn050AlgGel and Cu050AlgGel before and after 5000 charge/discharge cycles.

**Table S1.** Preparation conditions of MXAlgGel polymer electrolytes. Steps 1, 2, and 3 last for 1 hour, 1.5 hours, and 1.5 hours, respectively.

| **Step 1 (pure H_2_O)** | | | **Step 2 (gly 1:4 in H_2_O)** | | | **Step 3 (gly 1:4 in H_2_O)** | **Sample** |
| --- | --- | --- | --- | --- | --- | --- | --- |
| **MnCl_2_** | **CuCl_2_** | **LiCl** | **MnCl_2_** | **CuCl_2_** | **LiCl** |  |  |
| 1.00 M | | 0.00 M | 1.00 M | | 0.00 M |  | **Mn100AlgGel** |
| 0.75 M | | 0.25 M | 0.75 M | | 0.25 M |  | **Mn075AlgGel** |
| 0.50 M | | 0.50 M | 0.50 M | | 0.50 M |  | **Mn050AlgGel** |
| 0.25 M | | 0.75 M | 0.25 M | | 0.75 M |  | **Mn025AlgGel** |
| 0.00 M | | 1.00 M | 0.00 M | | 1.00 M |  | **LiAlgGel** |
| 1.00 M | | 0.00 M | 1.00 M | | 0.00 M |  | **Cu100AlgGel** |
| 0.75 M | | 0.25 M | 0.75 M | | 0.25 M |  | **Cu075AlgGel** |
| 0.50 M | | 0.50 M | 0.50 M | | 0.50 M |  | **Cu050AlgGel** |
| 0.25 M | | 0.75 M | 0.25 M | | 0.75 M |  | **Cu025AlgGel** |
| - | | - | 0.00 M | | 0.00 M |  | **AlgGel** |

**
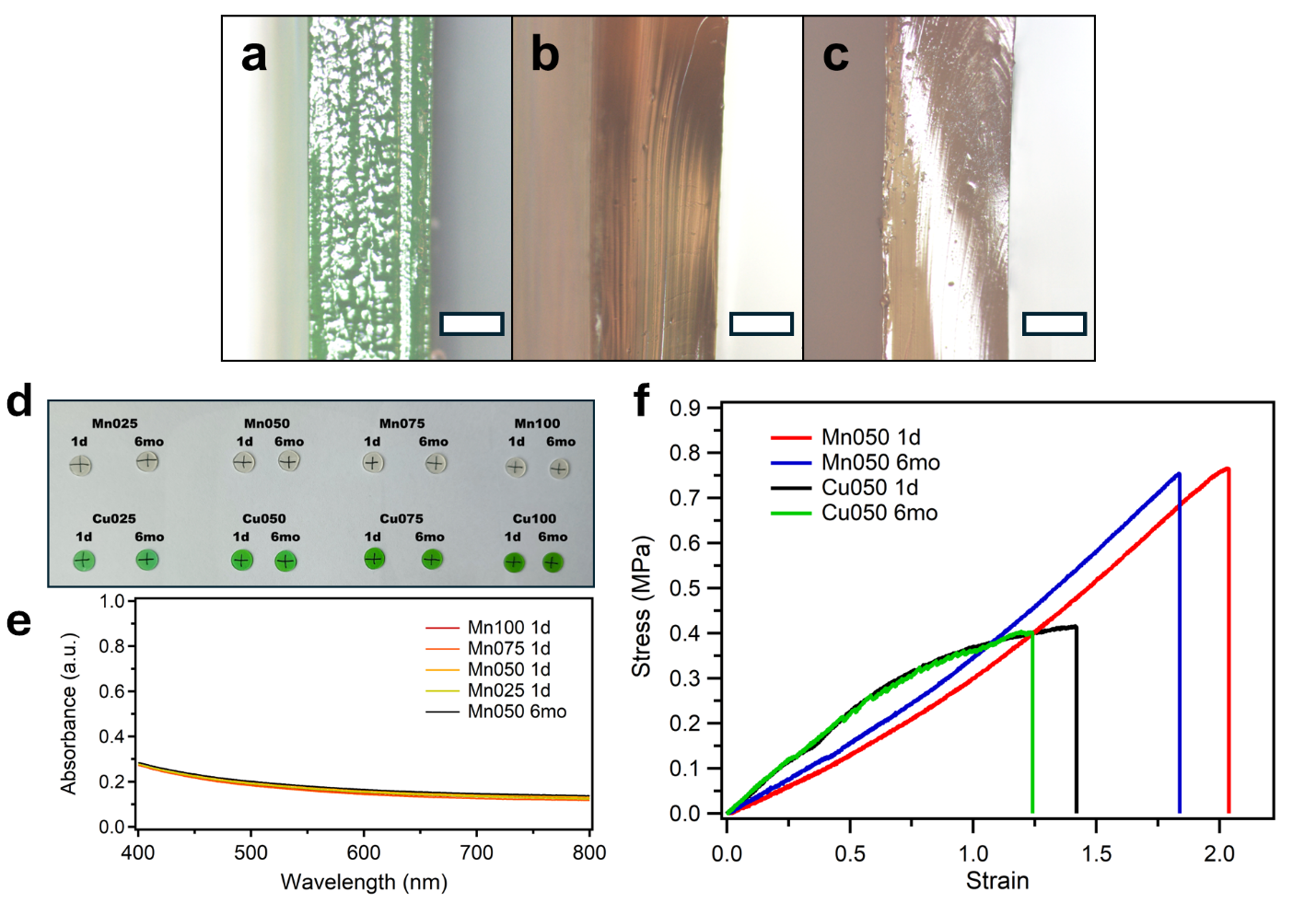
**

**Figure S1.** Optical microscopy side-view of a) Cu050AlgGel, b) LiAlgGel, and c) Mn050AlgGel for thickness evaluation. The lateral scale bar corresponds to 300 μm. d) Photographs of Mn- and Cu-crosslinked AlgGel samples after 1 day and 6 months of storage under ambient conditions, showing stable macroscopic appearance and minimal visual changes over time. A higher lithium concentration reduces the green colour intensity in CuXAlgGel, while MnXAlgGel remain visually unchanged. e) UV–vis absorbance spectra of Mn-crosslinked AlgGel samples after 1 day and 6 months, indicating excellent optical stability and the absence of significant aging-related changes in light transmittance. f) Stress–strain traction curves of Mn050AlgGel and Cu050AlgGel samples recorded after 1 day and 6 months, demonstrating retained mechanical properties with negligible degradation. Young’s modulus (YM) and Ultimate tensile strength (UTS) obtained are reported in Table S2.

**Table S2.** Young’s modulus (YM) and Ultimate tensile strength (UTS) of Mn050AlgGel 1d/6mo and Cu050AlgGel 1d/6mo. YM was evaluated in the 0.0 - 0.2 strain range.

| **Sample** | **Young’s modulus (MPa)** | **Ultimate tensile strength (MPa)** |
| --- | --- | --- |
| **Mn050 1d** | 0.24 ± 0.05 | 0.80 ± 0.06 |
| **Mn050 6mo** | 0.30 ± 0.04 | 0.75 ± 0.09 |
| **Cu050 1d** | 0.42 ± 0.07 | 0.43 ± 0.02 |
| **Cu050 6mo** | 0.50 ± 0.04 | 0.42 ± 0.02 |

**Table S3.** General properties of the AlgGel-based organohydrogels: thickness; water, salt, glycerol (Gly) and alginate-gelatin polymer (Polym) content; Thermal degradation onset (T_deg_). The error associated with T_deg_ is ± 2 °C. Further information on salt composition is reported in **Table S3**.

| **Sample** | **Thickness (μm)** | **Water (wt/wt%)** | **Salt**  **(wt/wt %)*** | **Gly (wt/wt%)** | **Polym**  **(wt/wt%)** | **T_deg_ (°C)** |
| --- | --- | --- | --- | --- | --- | --- |
| Mn100 | 600 ± 25 | 18.1 ± 0.5 | 5.2 ± 0.1 | ~ 58 | ~ 20 | 193 |
| Mn075 | 626 ± 35 | 17.4 ± 0.5 | 3.9 ± 0.2 | ~ 60 | ~ 20 | 192 |
| Mn050 | 608 ± 32 | 17.5 ± 0.5 | 3.0 ± 0.2 | ~ 61 | ~ 20 | 196 |
| Mn025 | 624 ± 15 | 16.7 ± 0.5 | 2.4 ± 0.1 | ~ 61 | ~ 20 | 195 |
| Li | 619 ± 22 | 15.1 ± 0.5 | 0.8 ± 0.1 | ~ 64 | ~ 20 | 199 |
| Cu100 | 610 ± 31 | 16.7 ± 0.5 | 5.2 ± 0.1 | ~ 58 | ~ 20 | 179 |
| Cu075 | 597 ± 19 | 17.8 ± 0.5 | 4.3 ± 0.3 | ~ 58 | ~ 20 | 183 |
| Cu050 | 604 ± 28 | 17.9 ± 0.5 | 2.6 ± 0.2 | ~ 58 | ~ 20 | 187 |
| Cu025 | 616 ± 20 | 19.6 ± 0.5 | 1.9 ± 0.1 | ~ 58 | ~ 20 | 190 |
| AlgGel | 592 ± 36 | 14.5 ± 0.5 | - | ~ 65 | ~ 20 | 195 |

**
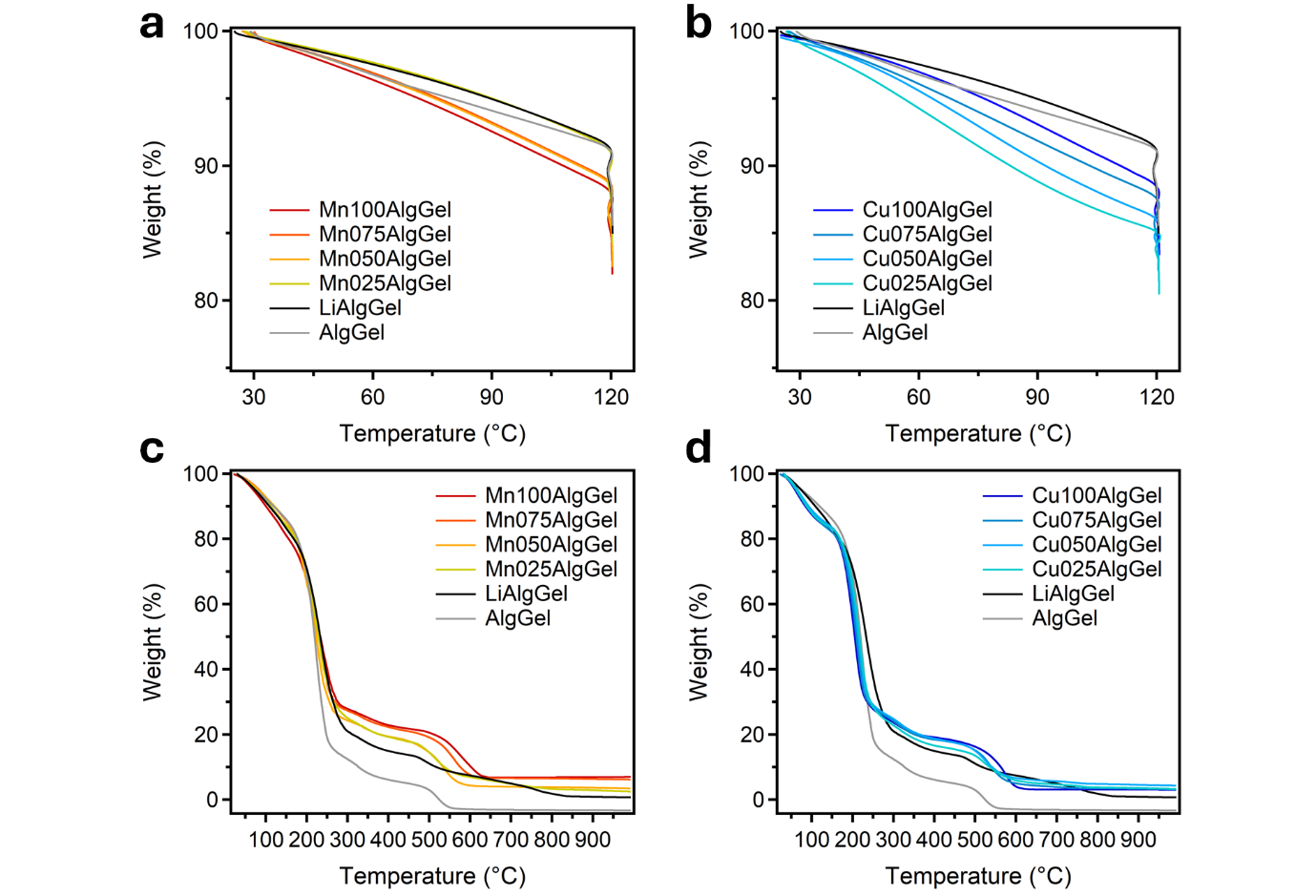
**

**Figure S2.** a, b) TGA thermograms for AlgGel-based organohydrogels’ water content determination. c, d) TGA thermograms for degradative profile evaluation.

**Table S4.** Experimental metal ions’ content of the AlgGel-based organohydrogels determined through ICP-AES.

| **Sample** | **Na^+^ (wt/wt%)** | **Cu^2+^ (wt/wt%)** | **Mn^2+^ (wt/wt%)** | **Li^+^ (wt/wt%)** | **nLi^+^/nM^2+^** |
| --- | --- | --- | --- | --- | --- |
| Mn100AlgGel | - | - | 5.2 ± 0.1 | - | 0 |
| Mn075AlgGel | - | - | 3.8 ± 0.1 | 0.11 ± 0.1 | 0.24 ± 0.02 |
| Mn050AlgGel | - | - | 2.8 ± 0.1 | 0.27 ± 0.1 | 0.77 ± 0.02 |
| Mn025AlgGel | - | - | 1.9 ± 0.1 | 0.49 ± 0.1 | 2.09 ± 0.02 |
| LiAlgGel | - | - | - | 0.75 ± 0.1 | - |
| Cu100AlgGel | - | 5.2 ± 0.1 | - | - | 0 |
| Cu075AlgGel | - | 4.2 ± 0.1 | - | 0.11 ± 0.1 | 0.25 ± 0.02 |
| Cu050AlgGel | - | 2.5 ± 0.1 | - | 0.15 ± 0.1 | 0.56 ± 0.02 |
| Cu025AlgGel | - | 1.7 ± 0.1 | - | 0.23 ± 0.1 | 1.28 ± 0.02 |
| AlgGel | 0.10 ± 0.1 | - | - | - | - |

**Table S5.** SAXS fitting parameters for the investigated AlgGel-based organohydrogels.

| **Sample** | **Scale** | **bkg** | **A** | **B** | **ξ (nm)** | **n** | **m** |
| --- | --- | --- | --- | --- | --- | --- | --- |
| Mn100 | 0.82 | 0.0205 | 9.67 ∙ 10^-7^ | 1.50 | 6.3 | 3.29 | 3.27 |
| Mn075 | 0.83 | 0.0213 | 3.47 ∙ 10^-6^ | 1.72 | 5.9 | 3.06 | 3.28 |
| Mn050 | 0.82 | 0.0179 | 3.53 ∙ 10^-6^ | 4.69 | 9.4 | 3.04 | 3.01 |
| Mn025 | 0.83 | 0.0117 | 1.01 ∙ 10^-5^ | 6.48 | 14.7 | 2.87 | 3.07 |
| Li | - | - | - | - | - | - | - |
| Cu100 | 0.83 | 0.0102 | 8.24 ∙ 10^-6^ | 0.70 | 3.9 | 2.26 | 2.91 |
| Cu075 | 0.82 | 0.0059 | 1.62 ∙ 10^-5^ | 0.52 | 3.8 | 2.13 | 2.79 |
| Cu050 | 0.82 | 0.0082 | 3.74 ∙ 10^-5^ | 0.73 | 3.6 | 2.01 | 2.64 |
| Cu025 | 0.80 | 0.0051 | 4.17 ∙ 10^-5^ | 0.63 | 3.5 | 2.01 | 2.61 |
| AlgGel | - | - | - | - | - | - | - |

**
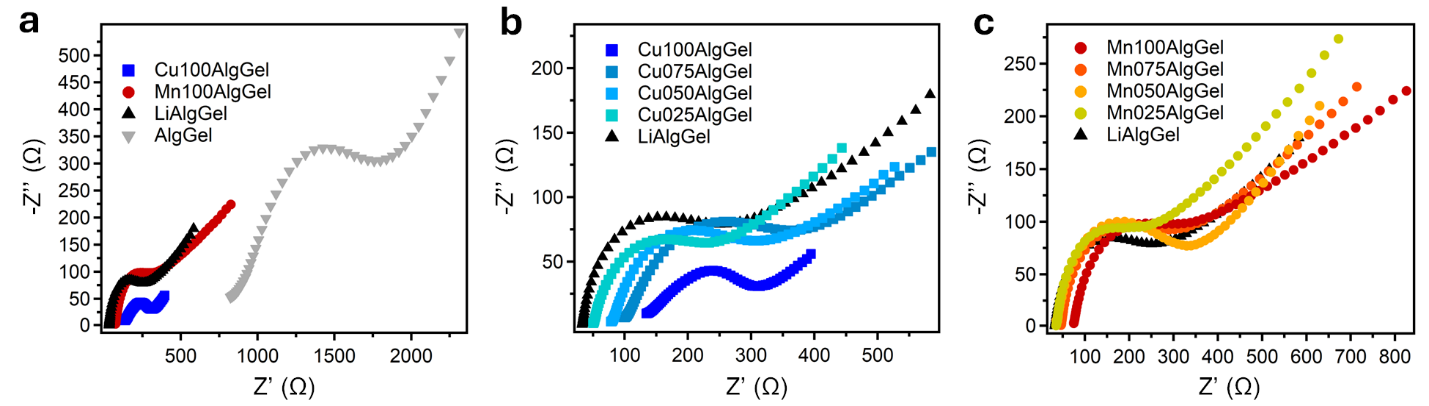
**

**Figure S3.** a) Nyquist plots of Cu100AlgGel, Mn100AlgGel, LiAlgGel and AlgGel. Nyquist plots of b) Cu100AlgGel, Cu075AlgGel, Cu050AlgGel, Cu025AlgGel and LiAlgGel and c) Mn100AlgGel, Mn075AlgGel, Mn050AlgGel, Mn025AlgGel and LiAlgGel.

**Table S6.** Bulk resistance (R_b_) and charge transfer resistance (R_ct_) values for Mn100AlgGel, Mn075AlgGel, Mn050AlgGel, Mn025AlgGel, LiAlgGel, Cu100AlgGel, Cu075AlgGel, Cu050AlgGel, Cu025AlgGel and AlgGel.

| **Sample** | **R_b_ (Ω)** | **R_ct_ (Ω)** | **σ (S/m)** |
| --- | --- | --- | --- |
| Mn100AlgGel | 73.3 | 296.4 | 1.64 |
| Mn075AlgGel | 46.0 | 289.7 | 2.72 |
| Mn050AlgGel | 40.2 | 282.3 | 3.02 |
| Mn025AlgGel | 36.8 | 243.1 | 3.39 |
| LiAlgGel | 32.7 | 238.1 | 3.79 |
| Cu100AlgGel | 119.0 | 239.9 | 1.03 |
| Cu075AlgGel | 94.1 | 299.8 | 1.27 |
| Cu050AlgGel | 82.8 | 242.4 | 1.46 |
| Cu025AlgGel | 52.5 | 186.7 | 2.35 |
| AlgGel | 836.4 | 1138.5 | 0.14 |


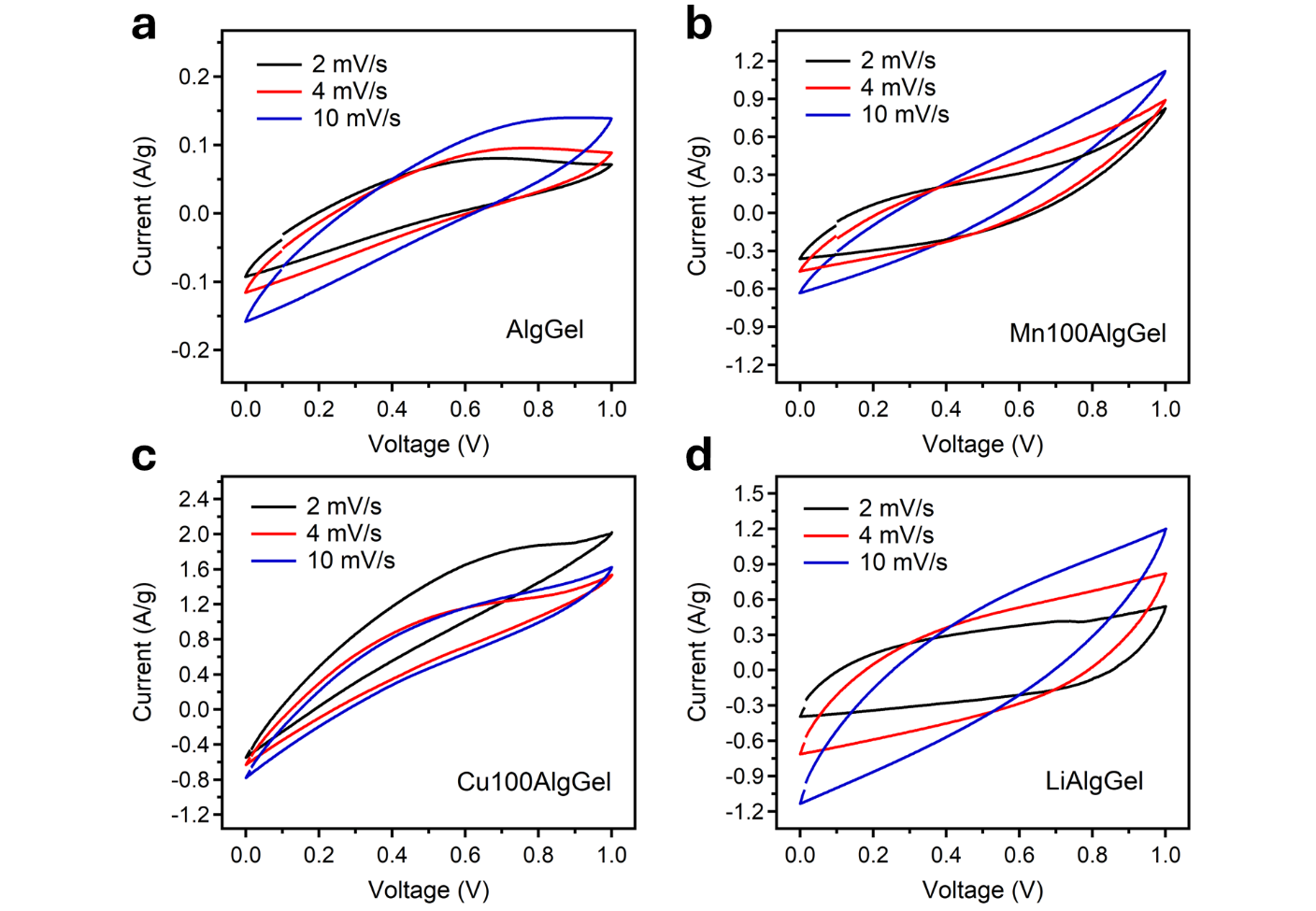


**Figure S4.** CV curves of a) AlgGel, b) Mn100AlgGel, c) Cu100AlgGel, and d) LiAlgGel in supercapacitors at different scan rates (*i.e.,* 2 mV/s, 4 mV/s and 10 mV/s).
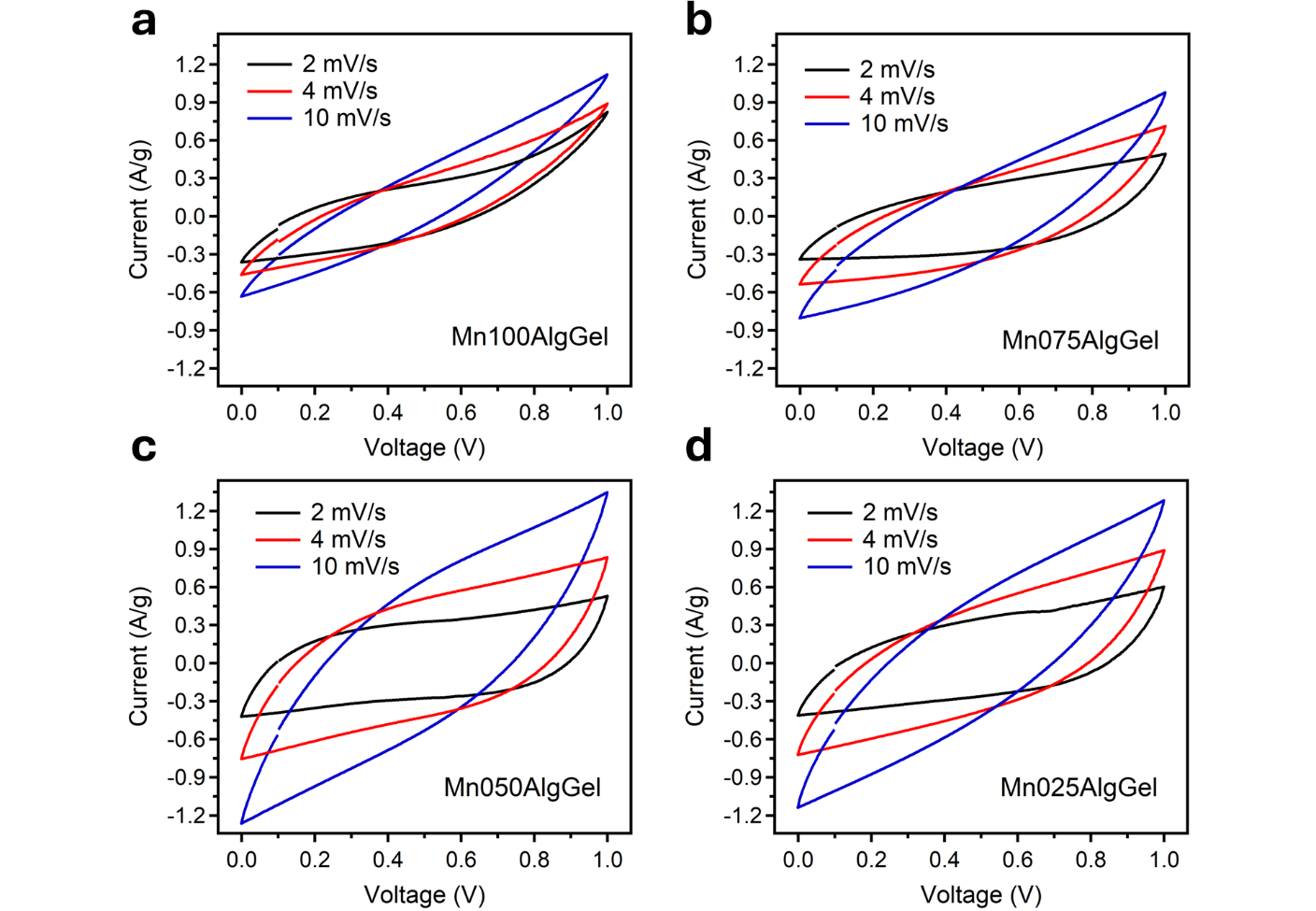


**Figure S5.** CV curves of a) Mn100AlgGel, b) Mn075AlgGel, c) Mn050AlgGel, and d) Mn025AlgGel in supercapacitors at different scan rates (*i.e.,* 2 mV/s, 4 mV/s and 10 mV/s).


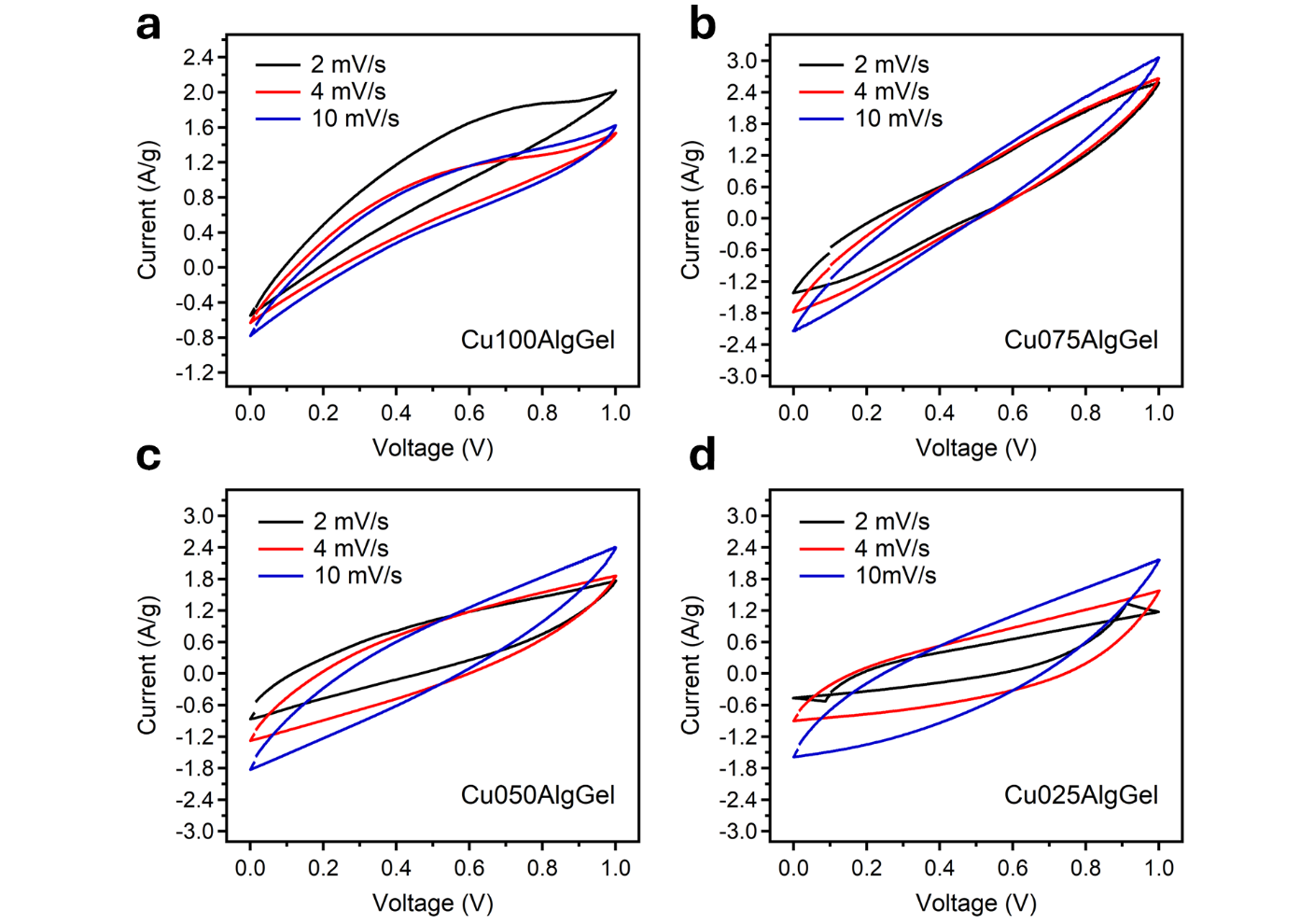


**Figure S6.** CV curves of a) Cu100AlgGel, b) Cu075AlgGel, c) Cu050AlgGel, and d) Cu025AlgGel in supercapacitors at different scan rates (*i.e.,* 2 mV/s, 4 mV/s, 10 mV/s).


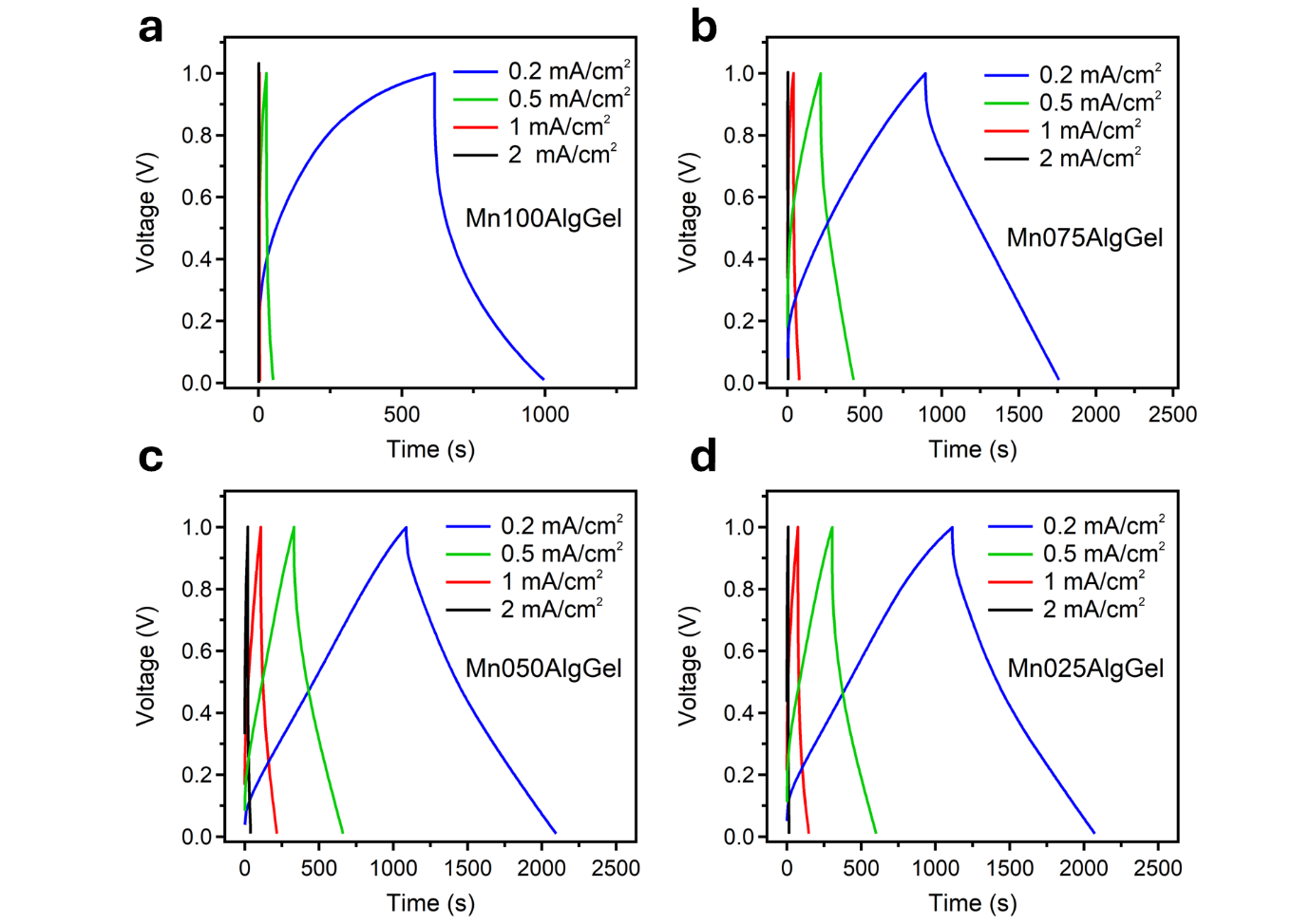


**Figure S7.** GCD curves of a) Mn100AlgGel, b) Mn075AlgGel, c) Mn050AlgGel, and d) Mn025AlgGel at different current densities (*i.e.,* 0.2 mA/cm^2^, 0.5 mA/cm^2^, 1.0 mA/cm^2^, 2.0 mA/cm^2^).


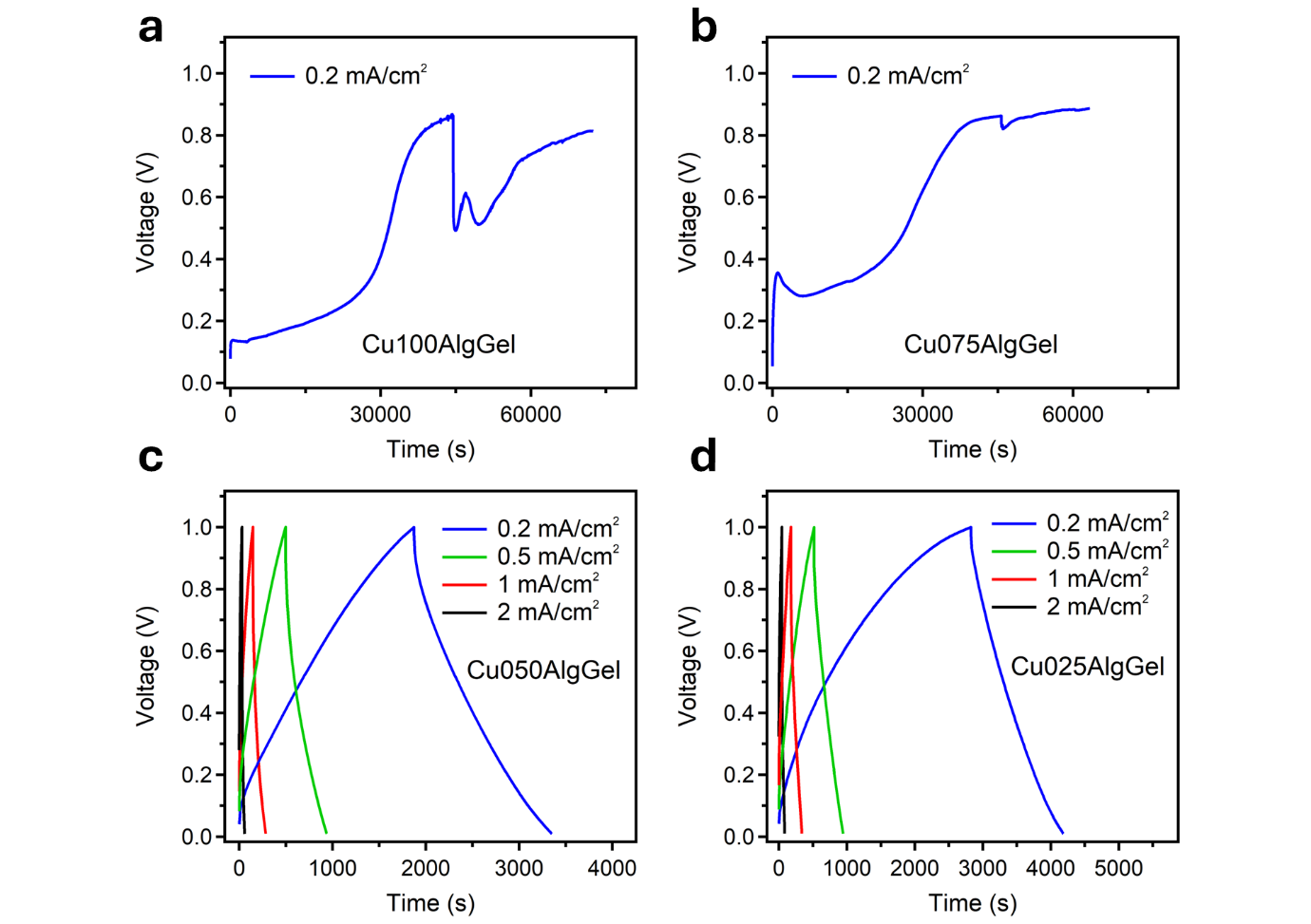


**Figure S8.** GCD curves of a) Cu100AlgGel, b) Cu075AlgGel, c) Cu050AlgGel, and d) Cu025AlgGel at different current densities (*i.e.,* 0.2 mA/cm^2^, 0.5 mA/cm^2^, 1.0 mA/cm^2^, 2.0 mA/cm^2^).


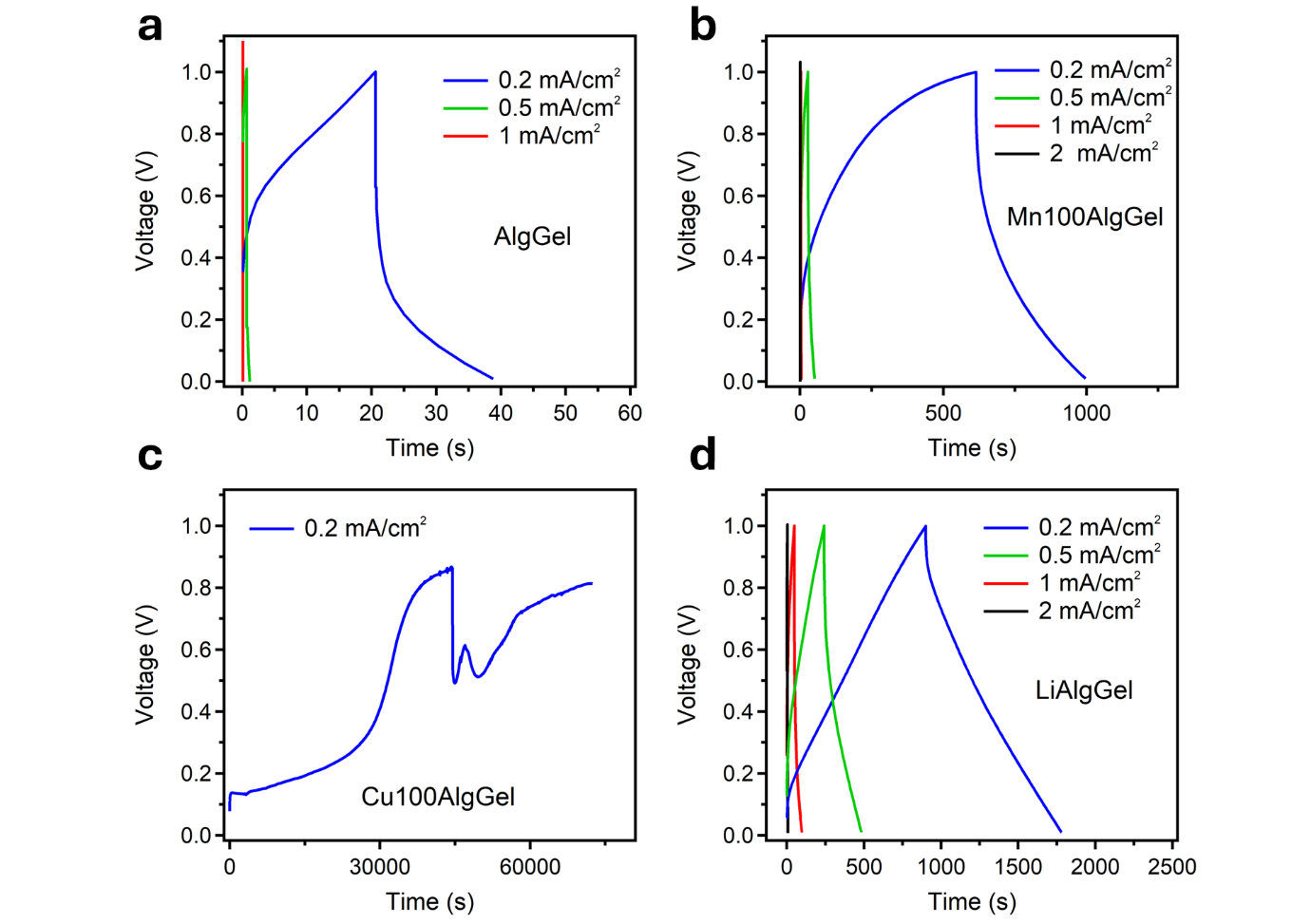


**Figure S9.** GCD curves of a) AlgGel, b) Mn100AlgGel, c) Cu100AlgGel, and d) LiAlgGel at different current densities (*i.e.,* 0.2 mA/cm^2^, 0.5 mA/cm^2^, 1.0 mA/cm^2^, 2.0 mA/cm^2^).

**Table S7.** Areal and specific capacitances of Mn050AlgGel, Cu050AlgGel and LiAlgGel at different current densities.

|  | **Mn050AlgGel** | **Cu050AlgGel** | **LiAlgGel** |
| --- | --- | --- | --- |
| **Areal capacitance (mF/cm^2^) at:** |  | | |
| 0.2 mA/cm^2^ | 394.2 ± 18.4 | 591.8 ± 1.8 | 339.3 ± 8.4 |
| 0.5 mA/cm^2^ | 329.8 ± 0.4 | 441.5 ± 1.3 | 240.7 ± 0.3 |
| 1.0 mA/cm^2^ | 215.0 ± 0.7 | 274.8 ± 3.0 | 96.8 ± 1.5 |
| 2.0 mA/cm^2^ | 78.2 ± 1.6 | 107.6 ± 3.8 | 12.8 ± 0.5 |
| **Specific capacitance (F/g) at:** |  | | |
| 0.10 A/g | 39.4 ± 1.8 | 59.2 ± 0.2 | 33.9 ± 0.9 |
| 0.25 A/g | 33.0 ± 0.1 | 44.2 ± 0.2 | 24.1 ± 0.1 |
| 0.50 A/g | 21.5 ± 0.1 | 27.5 ± 0.3 | 9.7 ± 0.2 |
| 1.00 A/g | 7.9 ± 0.2 | 10.8 ± 0.4 | 1.3 ± 0.1 |


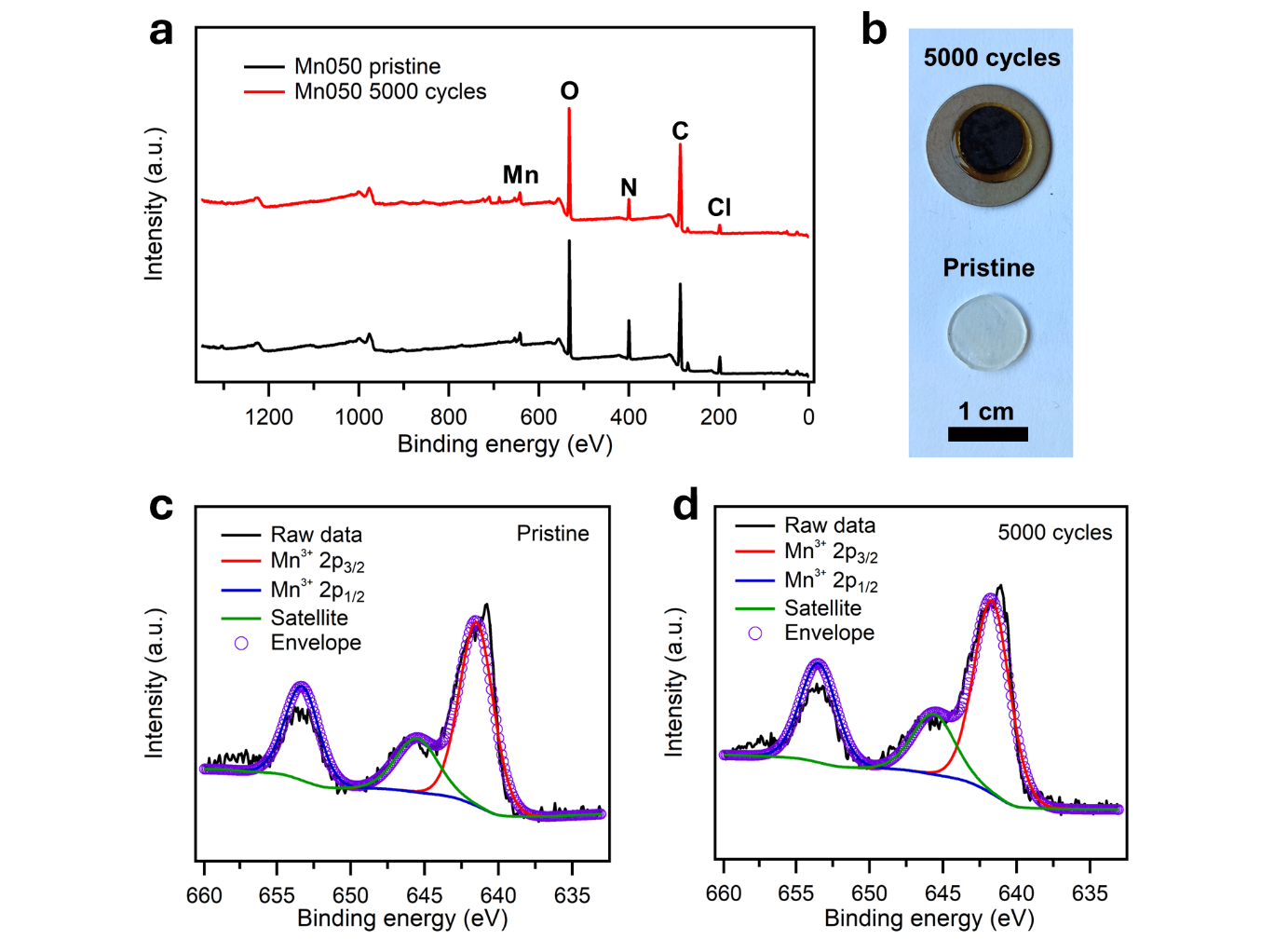


**Figure S10.** a) XPS survey spectra of Mn050AlgGel in the pristine state and after 5000 charge/discharge cycles show no significant change in elemental composition. b) Photographs of the gels before and after cycling indicate that the macroscopic structure and appearance remain intact, with minor darkening due to electrode contact. High-resolution Mn 2p spectra of the pristine (c) and cycled (d) Mn050AlgGel samples reveal signals characteristic of Mn^3+^, suggesting partial surface oxidation of Mn^3+^ during sample preparation or measurement. The near-identical profiles before and after cycling confirm that the Mn oxidation state remains stable and no additional redox changes occur during operation. These findings, supported by quantitative XPS data presented in Table S8, confirm the chemical and structural stability of the Mn^2+^-crosslinked network during electrochemical operation.


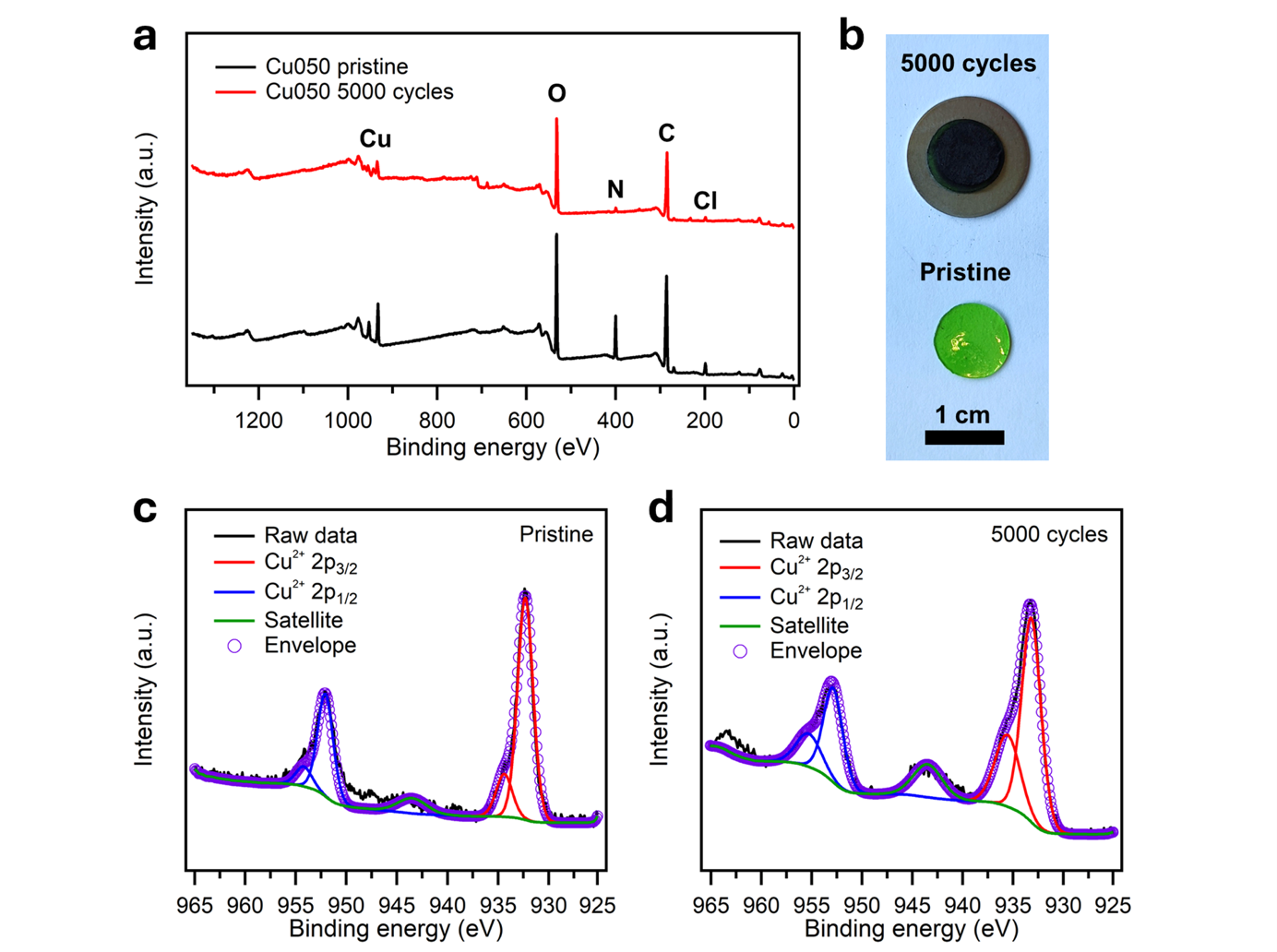


**Figure S11.** a) XPS survey spectra of Cu050AlgGel in the pristine state and after 5000 charge/discharge cycles reveal no significant changes in elemental composition. b) Photographs of the gels before and after cycling show preserved macroscopic appearance, with minor darkening due to electrode contact. High-resolution Cu 2p spectra of pristine (c) and cycled (d) gels confirm the retention of Cu oxidation state, as evidenced by the consistent shape and intensity of Cu 2p_3/2_, Cu 2p_1/2_, and satellite features. The oxidation state and coordination environment remain unaltered after cycling. These findings, supported by quantitative XPS data presented in Table S8, confirm the chemical and structural stability of the Cu^2+^-crosslinked network during electrochemical operation.

**Table S8.** Elemental composition of Mn050AlgGel and Cu050AlgGel before and after 5000 charge–discharge cycles. Results (with the related reference peak) are presented in atomic percentage.

| **Peak** | **Mn050 Pristine** | **Mn050 5000 cycles** | **Cu050 Pristine** | **Cu050 5000 cycles** |
| --- | --- | --- | --- | --- |
| C1s | (56.4 ± 0.1) % | (62.2 ± 0.2) % | (57.3 ± 0.2) % | (61.5 ± 0.2) % |
| O1s | (26.5 ± 0.2) % | (28.1 ± 0.2) % | (27.6 ± 0.1) % | (31.5 ± 0.1) % |
| N1s | (12.2 ± 0.2) % | (6.3 ± 0.1) % | (10.5 ± 0.2) % | (2.7 ± 0.1) % |
| Cl2p | (3.5 ± 0.3) % | (1.9 ± 0.2) % | (2.4 ± 0.2) % | (1.2 ± 0.1) % |
| Mn2p | (1.4 ± 0.1) % | (1.5 ± 0.1) % | - | - |
| Cu2p | - | - | (2.2 ± 0.1) % | (3.0 ± 0.1) % |

**
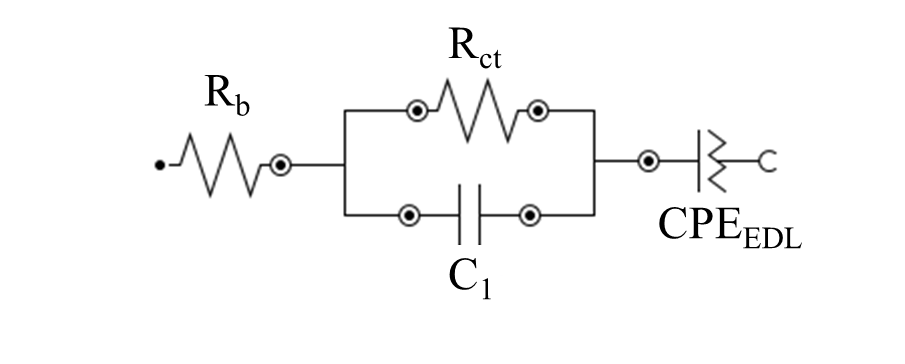
**

**Figure S12.** The equivalent electric circuit models used for fitting the Nyquist plots. R_b_: bulk resistance; R_ct_: charge transfer resistance; C_1_: capacitance element; CPE_EDL_: constant phase element representing the electrical double layer capacitance (EDLC).

**Table S9.** Summary of the state-of-the-art electrochemical performance of organohydrogel electrolytes for energy storage applications.

| **Ref** | **Electrodes** | **Electrolyte** | **Capacitance** | **Energy density** | **Power density** | **Capacitance retention (%)** |
| --- | --- | --- | --- | --- | --- | --- |
| This work | AC//AC | Mn050AlgGel | 394.2 mF/cm^2^ at 0.2 mA/cm^2^  39.4 F/g at 0.1 A/g | 54.8 μWh/cm^2^  5.5 Wh/kg | 1994.9 μW/cm^2^  192.7 W/kg | 88.3  (5000 cycles) |
| This work | AC//AC | Cu050AlgGel | 591.8 mF/cm^2^ at 0.2 mA/cm^2^  59.2 F/g at 0.1 A/g | 82.2 μWh/cm^2^  8.2 Wh/kg | 1957.8 μW/cm^2^  196.5 W/kg | 85.9  (5000 cycles) |
| 1 | Gr//Gr | SA/PAAm/  DMSO/KCl | 10.61 mF/cm^2^  at 2 mV/s | ~ 0.36 μWh/cm^2^ | ~ 6 μW/cm^2^ | 98.66  (10000 cycles) |
| 2 | AC//AC | Starch/PVA/  DMSO/CaCl_2_ | 156.50 mF/cm^2^  at 1 mA/cm^2^ | 21.74 μWh/cm^2^ | 526.29 μW/cm^2^ | 82.2  (8000 cycles) |
| 3 | AC//AC | Starch/PVA/  Gly/CaCl_2_ | 107.2 mF/cm^2^ at 1 mA/cm^2^ | 14.9 mWh/cm^2^ | 0.25 mW/cm^2^ | 84.5  (3000 cycles) |
| 4 | AC//AC | PVA/SA/  PEG/NaCl | 103.6 mF/cm^2^ at 2 mA/cm^2^ | 14.39 μWh/cm^2^ | 1 mW/cm^2^ | 84.3  (6000 cycles) |
| 5 | AC-CNT//  AC-CNT | PVA/P(AM-AMPS)/  Gly/Laponite/Na_2_SO_4_ | 186.1 mF/cm^2^ at 1 mA/cm^2^ | Not provided | Not provided | Not provided |
| 6 | Gly-Mo-PVA-PPy// Gly-Mo-PVA-PPy | PVA/(Gly-Mo)/  PPy/ H_2_SO_4_ | 140.75 mF/cm^2^ at 1 mA/cm^2^ | Not provided | Not provided | Not provided |
| 7 | CC-PANI//  CC-PANI | Amylopectin (AP)/Acrylic acid (AA)/acrylamide (AM)/PEG/Gly/KCl | 14.30 mF/cm^2^ at 0.2 mA/cm^2^ | Not provided | Not provided | Not provided |
| 8 | PANI//  PANI | PVA/Alg/CaCl_2_/  Gly(/EG/sorbitol) | 140.75 mF/cm^2^ at 1 mA/cm^2^ | Not provided | Not provided | Not provided |
| 9 | Ag@SWCNT// Ag@SWCNT | Regenerated cellulose/  EG/NaCl | 66.7 mF/cm^2^ at 0.67 mA/cm^2^ | 9.3 μWh/cm^2^ | 334.8 mW/cm^2^ | ~ 80  (1000 cycles) |
| 10 | CNTs//CNTs | PVA/EG/LiCl | ~ 17 mF/cm^2^ at ~ 0.5 mA/cm^2^ | Not provided | Not provided | 88.3  (5000 cycles) |
| 11 | AC//AC | PAMPS/PAAm/  EG/LiCl | 43 F/g at 1 A/g | 6.11 Wh/kg | 999.6 W/kg | 100  (10000 cycles) |
| 12 | AC//AC | HPC/PVA/  Gly/LiClO_4_ | 194.7 F/g at 2 A/g | 23.3 Wh/kg | 177.2 W/kg | 90.6  (5000 cycles) |
| 13 | CNTs//CNTs | MX-GO/  CNFs/SA/PVA | 5.4 F/g at 0.2 A/g | Not provided | Not provided | 98.2  (1500 cycles) |
| 14 | AC//AC | Gelatin/oxidized starch (OST)/Gly/ZnCl_2_ | 28.1 F/g at 0.5 A/g | 4.89 Wh/kg | 709 W/kg | 90.6  (2000 cycles) |
| 15 | AC//AC | PVA/PI/DMSO/LiCl | ~114 mF/cm^2^ at 1.67 mA/cm^2^ | 16.7 μWh/cm^2^ | 410.0 μW/cm^2^ | 95.3  (10000 cycles) |
| 16 | AC//AC | SPI/PVA/EG/  MgCl_2_/Na_3_Cit | 113.76 mF/cm^2^ at 1 mA/cm^2^ | 15.80 μWh/cm^2^ | 500 μW/cm^2^ | 81.6  (1000 cycles) |

Abbreviations: AC = activated carbon; Gr = graphene; SA = sodium alginate; CC = carbon cloth; poly(2-acrylamido-2-methylpropanesulfonic acid) =PAMPS; polyacrylamide = PAAm; PVA = Polyvinyl alcohol; Gly = glycerol; EG = ethylene glycol; PEG = polyethylene glycol; CNFs = cellulose nanofibrils; HPC = Hydroxypropylcellulose; SPI = soy protein isolate; PPy = polypyrrole; CNT = carbon nanotubes; PANI = polyaniline; CNTs = carbon nanotubes.

**
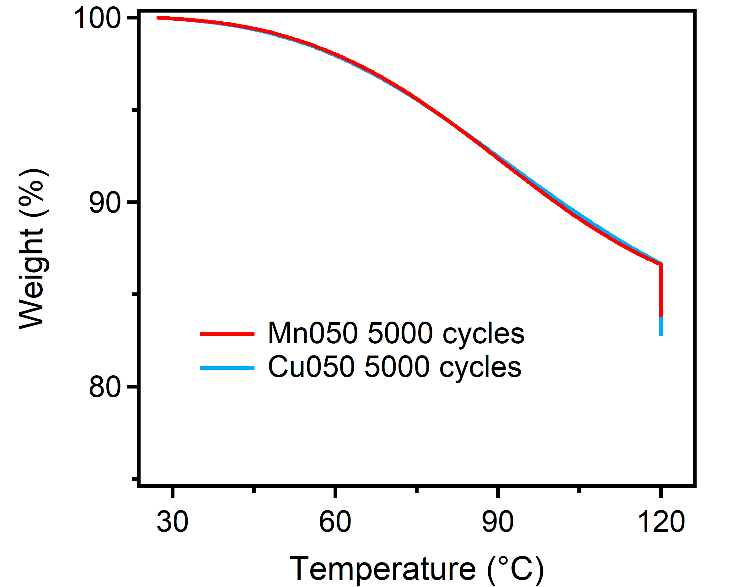
**

**Figure S13.** Thermogravimetric analysis (TGA) of Mn050AlgGel and Cu050AlgGel samples after 5000 charge/discharge cycles. The observed weight loss up to 120 °C, attributed to residual water content, shows a difference of less than 1.5% compared to their pristine counterparts. These results confirm the retention of water content after extended cycling. See also Figure S2 and Table S10 for comparison with pristine samples.

**Table S10.** Water content (wt/wt %) of Mn050AlgGel and Cu050AlgGel before and after 5000 charge/discharge cycles, as determined by TGA.

| **Sample** | **Water (wt/wt%)** |
| --- | --- |
| Mn050AlgGel | 17.5 ± 0.5 |
| Mn050AlgGel 5000 cycles | 16.0 ± 0.5 |
| Cu050AlgGel | 17.9 ± 0.5 |
| Cu050AlgGel 5000 cycles | 17.3 ± 0.5 |

**References**

[1] Y. Chen, L. Liu, Y. Huang, et al., Low-salt organohydrogel electrolytes for wide-potential-window flexible all-solid-state supercapacitors, 2024, *Appl. Energy*, *363*, 123100, https://doi.org/10.1016/j.apenergy.2024.123100

[2] L. He, J. Wang, S. Weng, & X. Jiang, A high-strength, environmentally stable, and recyclable starch/PVA organohydrogel electrolyte for flexible all-solid-state supercapacitor, 2023, *Carbohydr. Polym.*, *306*, 120587, https://doi.org/10.1016/j.carbpol.2023.120587

[3] J. Lu, J. Gu, O. Hu, et al., Highly tough, freezing-tolerant, healable and thermoplastic starch/poly(vinyl alcohol) organohydrogels for flexible electronic devices, 2021, *J. Mater. Chem. A*, *9*, 18406, https://doi.org/10.1039/D1TA04336F

[4] O. Hu, J. Lu, G. Chen, et al., An Antifreezing, Tough, Rehydratable, and Thermoplastic Poly(vinyl alcohol)/Sodium Alginate/Poly(ethylene glycol) Organohydrogel Electrolyte for Flexible Supercapacitors, 2021, *ACS Sustain. Chem. Eng.*, *9*, 9833, https://doi.org/10.1021/acssuschemeng.1c02464

[5] X. Jiang, S. Wei, & J. Wang, Preparation of Tough and Adhesive PVA/P(AM-AMPS)/Glycerol/Laponite/Na2SO4 Organohydrogels for All-Solid-State Supercapacitors and Self-Powered Wearable Strain Sensors, 2024, *ACS Appl. Mater. Interfaces*, *16*, 1380, https://doi.org/10.1021/acsami.3c13256

[6] Q. Xin, X. Chu, G. Yang, S. Liang, & J. Lin, An anti-freezing and conductive glycerol-Mo-based organohydrogel electrolyte for flexible supercapacitor, 2023, *Ionics*, *29*, 4275, https://doi.org/10.1007/s11581-023-05140-6

[7] H. Zhou, J. Lai, B. Zheng, et al., From Glutinous-Rice-Inspired Adhesive Organohydrogels to Flexible Electronic Devices Toward Wearable Sensing, Power Supply, and Energy Storage, 2022, *Adv. Funct. Mater.*, *32*, 2108423, https://doi.org/10.1002/adfm.202108423

[8] J. Chen, Q. Yu, D. Shi, et al., Tough and Antifreezing Organohydrogel Electrolyte for Flexible Supercapacitors with Wide Temperature Stability, 2021, *ACS Appl. Energy Mater.*, *4*, 9353, https://doi.org/10.1021/acsaem.1c01556

[9] C. Qin, & A. Lu, Flexible, anti-freezing self-charging power system composed of cellulose based supercapacitor and triboelectric nanogenerator, 2021, *Carbohydr. Polym.*, *274*, 118667, https://doi.org/10.1016/j.carbpol.2021.118667

[10] Q. Rong, W. Lei, J. Huang, & M. Liu, Low Temperature Tolerant Organohydrogel Electrolytes for Flexible Solid-State Supercapacitors, 2018, *Adv. Energy Mater.*, *8*, 1801967, https://doi.org/10.1002/aenm.201801967

[11] X. Li, D. Lou, H. Wang, X. Sun, J. Li, & Y.-N. Liu, Flexible Supercapacitor Based on Organohydrogel Electrolyte with Long-Term Anti-Freezing and Anti-Drying Property, 2020, *Adv. Funct. Mater.*, *30*, 2007291, https://doi.org/10.1002/adfm.202007291

[12] N. Lu, R. Na, L. Li, et al., Rational Design of Antifreezing Organohydrogel Electrolytes for Flexible Supercapacitors, 2020, *ACS Appl. Energy Mater.*, *3*, 1944, https://doi.org/10.1021/acsaem.9b02379

[13] K. Hu, Z. Zhao, Y. Wang, et al., A tough organohydrogel-based multiresponsive sensor for a triboelectric nanogenerator and supercapacitor toward wearable intelligent devices, 2022, *J. Mater. Chem. A* **2022**, *10*, 12092, https://doi.org/10.1039/D2TA01503J

[14] Y. Liang, Q. Song, Y. Chen, C. Hu, & S. Zhang, Stretch-Induced Robust Intrinsic Antibacterial Thermoplastic Gelatin Organohydrogel for a Thermoenhanced Supercapacitor and Mono-gauge-factor Sensor, 2023, *ACS Appl. Mater. Interfaces*, *15*, 20278, https://doi.org/10.1021/acsami.3c02255

[15] Q. Zhou, A. Griffin, J. Qian, Z. Qiang, B. Sun, C. Ye, & M. Zhu, Mechanically Strong and Tough Organohydrogels for Wide Temperature Tolerant, Flexible Solid-State Supercapacitors, 2024, *Adv. Funct. Mater.*, *34*, 2405962, https://doi.org/10.1002/adfm.202405962

[16] M. Hu, Y. Deng, X. Qian, D. Ye, X. Jiang, & G. Xiao, One-pot preparation of strong, tough, frost-resistant and recyclable organohydrogels via Hofmeister effect and its application for electronic devices, 2024, *Eur. Polym. J.*, *221*, 113529, https://doi.org/10.1016/j.eurpolymj.2024.113529
